# The interaction of force and repetition on musculoskeletal and neural tissue responses and sensorimotor behavior in a rat model of work-related musculoskeletal disorders

**DOI:** 10.1186/1471-2474-14-303

**Published:** 2013-10-25

**Authors:** Mary F Barbe, Sean Gallagher, Vicky S Massicotte, Michael Tytell, Steven N Popoff, Ann E Barr-Gillespie

**Affiliations:** 1 Department of Anatomy and Cell Biology, Temple University School of Medicine, 3500 North Broad St, Philadelphia 19140, PA, USA; 2 Comprehensive NeuroAIDS Center, Temple University School of Medicine, Philadelphia, PA 19140, USA; 3 Department of Industrial and Systems Engineering, Auburn University, Auburn, Alabama 36849-5346, USA; 4 Department of Neurobiology and Anatomy, Wake Forest University School of Medicine, Winston-Salem, NC 27157-1010, USA; 5 College of Health Professions, Pacific University, Hillsboro, OR 97123, USA

**Keywords:** Overuse, Reaching and grasping task, Musculoskeletal disorders, Repetitive strain injury, Cytokines, HSP72, TGFB1

## Abstract

**Background:**

We examined the relationship of musculoskeletal risk factors underlying force and repetition on tissue responses in an operant rat model of repetitive reaching and pulling, and if force x repetition interactions were present, indicative of a fatigue failure process. We examined exposure-dependent changes in biochemical, morphological and sensorimotor responses occurring with repeated performance of a handle-pulling task for 12 weeks at one of four repetition and force levels: 1) low repetition with low force, 2) high repetition with low force, 3) low repetition with high force, and 4) high repetition with high force (HRHF).

**Methods:**

Rats underwent initial training for 4–6 weeks, and then performed one of the tasks for 12 weeks, 2 hours/day, 3 days/week. Reflexive grip strength and sensitivity to touch were assayed as functional outcomes. Flexor digitorum muscles and tendons, forelimb bones, and serum were assayed using ELISA for indicators of inflammation, tissue stress and repair, and bone turnover. Histomorphometry was used to assay macrophage infiltration of tissues, spinal cord substance P changes, and tissue adaptative or degradative changes. MicroCT was used to assay bones for changes in bone quality.

**Results:**

Several force x repetition interactions were observed for: muscle IL-1alpha and bone IL-1beta; serum TNFalpha, IL-1alpha, and IL-1beta; muscle HSP72, a tissue stress and repair protein; histomorphological evidence of tendon and cartilage degradation; serum biomarkers of bone degradation (CTXI) and bone formation (osteocalcin); and morphological evidence of bone adaptation versus resorption. In most cases, performance of the HRHF task induced the greatest tissue degenerative changes, while performance of moderate level tasks induced bone adaptation and a suggestion of muscle adaptation. Both high force tasks induced median nerve macrophage infiltration, spinal cord sensitization (increased substance P), grip strength declines and forepaw mechanical allodynia by task week 12.

**Conclusions:**

Although not consistent in all tissues, we found several significant interactions between the critical musculoskeletal risk factors of force and repetition, consistent with a fatigue failure process in musculoskeletal tissues. Prolonged performance of HRHF tasks exhibited significantly increased risk for musculoskeletal disorders, while performance of moderate level tasks exhibited adaptation to task demands.

## Background

Musculoskeletal disorders (MSDs) are a leading cause of long-term pain and physical disability world-wide [1-4], with diagnoses including tendinopathies, nerve compression syndromes, and muscular and joint disorders [5-8]. Musculoskeletal conditions are the second greatest cause of disability globally and have increased 45% worldwide, according to the 2010 Global Burden of Disease Study [4]. In 2011, MSDs accounted for 33% of all lost work time, workplace injuries and illnesses in the U.S. and a median of 11 days absence from work [2]. Occupationally related MSDs in the United States are associated with 130 million health care encounters and are estimated to cost over $50 billion annually [9]. In 2011, the number of occupational injuries involving days away from work due to hand and wrist injuries were 140,460 and 47,550, respectively, with incidence rates of 13.9 and 4.7 per 10,000 workers, respectively [2]. There is also high incidence of MSDs among military personnel, with MSDs classified as “inflammation/pain (overuse)” constituting 82% of these injuries, and upper extremity injuries comprising 14% of the total [10]. Variances in prevalence and anatomical location of bone stress fractures, for example, are reflective of differences in military branches, training programs, sports, duration of activity, degree of physical rigor, equipment, case definitions, methodologies, and gender (females have higher incidence) [11-14].

Many volitional [15-22] and non-volitional [19,23-31] animal models have been utilized for the investigation of the induction of MSDs in muscles, tendons, nerves, and bones. In these studies, tissue damage was exposure dependent, increasing with amount of force or cyclical loading, frequency or chronicity of loading, and recovery time between bouts of loading. For example, single stretch muscle contraction models that stretch muscles within physiological range do not show muscle damage or pronounced force deficits [32,33]. However, repeated stretching within physiological range can produce muscle damage [27,34], thereby supporting the concept that cumulative low amplitude contractions produce muscle damage [10]. Greater motor performance deficits occur with shorter compared to longer rest cycles between bouts of muscle contractions [26]. Chronic stretch-shortening contractions studies show that skeletal muscle adaptation (defined as remodeling with functional gains) can occur if the muscle is able to compensate to the increased demands of an activity, but that maladaptive muscles changes (subdegenerative or subnecrotic injury with low levels of persistent inflammation and loss of function) occur if muscles are not able to meet these demands [25,35]. In bone, failure of adaptative process is also a key factor in microdamage and fatigue fracture mechanisms [28].

MSDs often result from physical demands placed upon the musculoskeletal system and peripheral nerves in the workplace [8,36-38]. Although acute trauma may be a factor in some cases of MSDs, many occupational-related MSDs are the result of cumulative effects of smaller amplitude forces that occur with overtraining, overexertion, repetitive movements and activities, forceful actions, and prolonged static positioning [10]. Forceful exertions, repetitive motion, and non-neutral body postures have been identified as key risk factors [39]. Of particular interest in this article, is a possible interaction between the critical musculoskeletal risk factors of force and repetition. A recent systematic review of the occupational-related MSD epidemiology literature examined studies that tested for an interaction between these two risk factors [40]. Evidence of interaction was found in 10 of 12 epidemiologic studies. A consistent pattern of interaction was observed across a number of disorders, including carpal tunnel syndrome, tendinitis, epicondylitis, hand pain and low back disorders, with low force tasks demonstrating a small or modest increase in MSD risk with increased repetition, while high force tasks consistently exhibited an escalation in MSD risk, especially when combined with increased repetition [40]. The authors of that review provided a theoretical basis for the interaction between force and repetition, suggesting that this interaction pattern would be anticipated if musculoskeletal tissues incur damage as the result of fatigue failure with prolonged performance of occupational-related tasks.

After tissue damage, inflammatory cells infiltrate tissues, which, along with injured cells, produce inflammatory cytokines and other mediators that either exacerbate damage or assist in tissue repair [35,41,42]. Inflammation and histopathology are often congruent [43], and the magnitude of an inflammatory response to tissue insult or overuse appears to be reflective of the extent of injury in the tissues [42,44-46]. If tissues are damaged as the result of a fatigue failure process, as suggested previously in several studies [28,31,40,47-51], a specific pattern of interaction would be expected in the responses of inflammatory mediators in tissues exposed to one of four possible combinations of low and high force and repetition. A continuum of inflammatory responses would be expected, with low repetition low force (LRLF) tasks showing the least, and high repetition high force (HRHF) tasks showing the most. In contrast, low repetition high force (LRHF) and high repetition low repetition (HRLF) tasks would show intermediate inflammatory responses. A similar pattern of force x repetition interaction might be evident in adaptative/repair or degradative responses, which may be superimposed with inflammation, with continued performance of repetitive tasks, according to a previously hypothesized tissue tolerance model for MSDs [41] and tissue adaptation hypotheses [52,53]. The tissue tolerance model postulates that prolonged performance of low and moderate activities will lead to adaptation in involved tissues, while prolonged performance of high demand activities, such as the HRHF task, might lead to structural degradation. Such findings would be consistent with fatigue failure processes and Selye’s General Adaptation Syndrome, since the third stage of Selye’s model (the exhaustion stage) is a reappearance of tissue inflammation and catabolism in the course of chronic exposure to a stressor, due to a finite quantity of “adaptation energy” [46,53]. That said, there are many high-intensity exposure conditions (i.e. work hardening, wellness, sport specific training, and rehabilitative approaches/techniques) that do not lead to structural degradation. This may be due to differences in metabolic, tissue stress-related mRNA, or protein responses with repeated stressful work versus exercise training, as shown recently [54]. With occupational-related MSDs, a fatigue failure process has been observed, as described above [40]. However, it has yet to be examined methodically in multiple tissue types in a controlled animal study of occupational-related WSDs.

Our goal here was to test the above-predicted physiological responses (inflammation, degradation and adaptation) in a unique, operant behavior rat model of repetitive reaching and handle pulling in which we can examine these responses in several tissue types (muscle, tendon, bone, neural and serum), and in sensorimotor behaviors, after performance of tasks for 2 hours/day, 3 days/week, for 12 weeks, at one of the four different repetition and force levels described above. We used an operant model developed previously [22,55-58], in which rats reach forward using their whole forearm, into a portal that constrains their posture, for a handle located outside of the chamber, and then grasp the handle that is attached to a stationary force transducer, at a learned and defined reach rate and target force for a food reward. The grasp is isometric in type, but not pure, since it is an operant task and rats are free to alter their forearm and forepaw position to achieve success. We also examined tissue and sensorimotor responses at the end of an initial training period (week 0 of task performance) in which rats were learning the tasks in 10 min session/day, 5 days/week, for 4–6 weeks, in order to investigate if there were training-induced responses that might inform development of future preventative or early treatments. Some of these data have been published previously, such as portions of the LRHF data [55], HRLF data [59-61], and HRHF data [18,22,58,60,62]. We have indicated in the methods section which data has been published previously. To our knowledge, this is the first time that heat shock protein 72 (HSP72) levels in muscles and tendons, tendon levels of platelet derived growth factor (PDGF) and matrix metalloproteinase 2 (MMP), serum levels of CTX-1 (a bone degradation marker), and bone morphometry using micro-computerized tomography, have been examined in an operant animal model of upper extremity repetitive loading. Importantly, this is the first time that multiple behavioral and tissue analytes have been compared across all four repetition and force loading levels in a controlled animal study examining responses in multiple tissue types.

## Methods

### Animals and overview

All experiments were approved by the Temple University Institutional Animal Care and Use Committee and were in compliance with NIH guidelines for humane care and use of laboratory animals. A total of 275 young adult (14 weeks of age at onset of study) female Sprague–Dawley rats were randomly divided into 10 groups. There were four groups of rats that underwent training only for 10 min/day, 5 days/week, for 4–6 weeks, in an initial learning “training” period, in which the rats ramped upwards from naïve to being able to perform one of the four tasks by the final week of training: 0-week low repetition low force (LRLF; n = 35), 0-week high repetition low force (HRLF; n = 35), 0-week low repetition high force (LRHF; n = 21), or 0-week high repetition high force (HRHF; n = 45) rats. Trained-only rats were euthanized at the end of this initial learning period, a time point equivalent to week 0 of the task period. There were four more groups of rats that underwent the same training, and then went on to perform the tasks for 2 hours/day, 3 days/week, for a total of 12 weeks: 12-week LRLF (n = 18), 12-week HRLF, (n = 23), 12-week LRHF (n = 22), and 12-week HRHF (n = 28) rats. Results were compared to normal control rats (NC, n = 30) or food restricted control rats (FRC, n = 18) that were euthanized at age-matched time points as 12-week task rats.

Adult female rats were used for several reasons: (1) Human females have a higher incidence of work-related MSDs than males [63-65]; and (2) for inclusion of data from our past studies on female rats. Rats were housed in a central animal facility in separate cages with a 12-hour light: dark cycle and free access to water.

The animal numbers used for the various assays may differ across groups and by assay as these data are the result of 10 years of experimentation and not all data were collected at the same time. However, the assays and stains used in this study were performed by the same individuals and using the same companies and kits across the span of these experiments to reduce variability, or were assayed as a batch for the purpose of this study (e.g. heat shock protein (HSP72, a stress and repair protein), transforming growth factor beta 1 (TGFB1, a cytokine related to repair, although it may be fibrotic repair [66,67]), PDGFaa and bb (repair proteins), MMP2 (a tissue degradative enzyme), presence of activated macrophages within individual myofibers, and micro computerized tomography (microCT) to assay bone quality in the distal radius).

### Behavioral apparatuses

The sixteen behavioral apparatuses used were as previously described, and as shown in Additional file 1: Figure S1 and in the Additional file 2[22,58,60]. Briefly, animals reached through a shoulder height portal that constrained their posture, with the whole arm extended, and then grasped a handle located outside of the chamber wall that was attached to a stationary force transducer (Futek Advanced Sensor Technology, Irvine, CA) (Additional file 1: Figure S1 and Additional file 2). Load cells were interfaced with custom written Force-Lever software (version 1.03.02, Med Associates, St. Albans, VT). Auditory and light indicators cued reaching rates. If reach and force criteria (defined below and in Table 1) were met within a 5 second cueing period, a 45 mg food pellet was dispensed into a trough for the animal to lick up.

**Table 1:** Task parameters: target versus actual (Mean ± SEM reported)

		**LRLF**			**HRLF**			**LRHF**			**HRHF**	
	**Successful reps/min (n = 12)**	**All reps/min (n = 12)**	**Grasp force% MPF (n = 12)**	**Successful reps/min (n = 24)**	**All reps/min (n = 24)**	**Grasp force% MPF (n = 15)**	**Successful reps/min (n = 12)**	**All reps/min (n = 12)**	**Grasp force% MPF (n = 12)**	**Successful reps/min (n = 24)**	**All reps/min (n = 24)**	**Grasp force% MPF (n = 24)**
Target values	2	2	15 ± 5% MPF (a mean of 0.23 N)	4	4	15 ± 5% MPF (a mean of 0.23 N)	2	2	53 ± 5% MPF (a mean of 1.02 N)	4	4	53 ± 5% MPF (a mean of 1.02 N)
Week 1 actual values	2.01 ± 0.33	3.03 ± 0.12	15.78 ± 4.54	2.28 ± 0.34	6.48 ± 0.47	20.07 ± 1.61	1.31 ± 0.38	9.0 ± 0.68	41.90 ± 2.61	2.31 ± 0.52	10.8 ± 0.80	39.88 ± 2.22
Week 12 actual values	1.39 ± 0.06	3.38 ± 0.31	13.68 ± 0.89	2.06 ± 0.39	5.04 ± 0.45	14.95 ± 1.03	1.46 ± 0.25	5.79 ± 0.97	46.22 ± 6.24	2.89 ± 0.46	9.27 ± 0.64	48.64 ± 0.90

All Reps = all repetitions per minute, both successful and unsuccessful; Grasp Force =% maximum isometric pulling force on lever bar; HRLF = high repetition low force task: HRHF = high repetition high force task; LRLF = low repetition low force task; LRHF = low repetition high force task; MPF = average maximum isometric pulling force for these young adult rats, which was determined to be 1.93 Newtons on the last day of training by a subset of rats; n = number of rats analyzed for this data; N = Newtons. Week 1 rather than week 0 is reported as this was the first week that the rats actually performed the tasks for 2 hours/day, 3 days/week.

### Initial training to learn the tasks

All rats except for NC rats were food-restricted for a short period (no more than 7 days) to 85-95% of their naive weight to initiate interest in the food pellets [57]. After that first week, rats were given extra rat chow, weighed weekly, and their food was adjusted weekly to maintain 95% body weight of age-matched controls, until euthanasia. All but 18 of the food restricted rats were randomly divided into one of four groups that went through an initial training period to learn of the four different operant tasks for 10 min/day, 5 days/week, for 4–6 weeks. The remaining 18 food restricted rats did not undergo this training and became food restricted control rats (FRC). During this period, rats were trained to perform the reaching and handle-pulling tasks at the appropriate reach rate and force requirements for a particular task, as previously described [22,57], and as defined in Table 1. The rats learned to perform one of the four tasks during this training period, reaching the target reach rate and force requirements for their respective group in their final week of training. The lower demand tasks took less time to learn (4 weeks) than the higher demand tasks (6 weeks). After this training period, cohorts of trained rats went on to perform one of the four task regimens for 12 weeks. The remaining trained-only rats “0-week rats” were euthanized immediately after training to examine their tissues for potential training effects.

### LRLF, HRLF, LRHF, HRHF task regimens

After the training period, task rats went on to perform one of the four task regimens for 2 hrs/day, 3 days/wk for 12 weeks. Daily task sessions were divided into 4, 0.5-hr sessions separated by 1.5 hrs in order to avoid satiation. Rats were cued using auditory and light cues to reach at target rates of 2 or 4 reaches/min, for low repetition or high repetition, respectively (Table 1; Additional file 1: Figure S1 1A,F and Additional file 2). The task rats reached forward into a portal, extended their forearm, grabbed a handle and then exerted a target isometric pull for at least 50 ms at a force effort of either 15% (0.23 Newtons) or 53% (1.02 Newtons) of their average maximum pulling force (± 5%), for low force or high force, respectively, as appropriate for their group, for a food reward (Table 1; Additional file 1: Figure S1). Because the inherent nature of our task is voluntary, the rats were not prevented from reaching more frequently than cued, or from exerting a pull that was at a higher or lower force than their target force. Thus, the animals were allowed to self-regulate their participation in task performance. However, if they either undershot the minimum criteria (−5%) or overshot the maximum criteria (+5%), no food reward was delivered (which is considered an unsuccessful pull, as described in more detail previously [60]). These criteria had to be met within a 5 second window initiated every 15 (high repetition) or 30 seconds (low repetition), and held the handle with the correct force for 50 ms (Additional file 1: Figure S1C). If these criteria are met, than rats receive a food reward deposited into a food trough (Additional file 1: Figure S1D), and this is considered as a successful reach. Table 1 lists the mean number of both successful and unsuccessful reaches per group.

### Estimation of actual reach rate and voluntary grasp force

Force lever data were recorded continuously during each task session for later calculation of reach performance data (reach rate and voluntary grasp force) manually and via an automated script (MatLab; Mathworks, Natick, MA), as described previously [60]. Force lever data were obtained, including reach rate (reaches/min), all reaches versus successful reaches, and voluntary grasp force, and calculated from subsets of rats in weeks 1 and 12, as shown in Table 1. The end of week 1 was used as the baseline for reach performance variables since that was the first week that task rats actually performed the task regimens. Part of the HRLF, LRHF and HRHF data for reach and grasp force has been reported previously [22,55,60].

### Grip strength (Reflexive) analysis

Reflexive grip strength in the preferred the reach limb was tested using a grip strength meter for rodents (Stoelting, Wood Dale, IL, USA), as previously described [57] in: 0- and 12-week LRLF (n = 35 and 12, respectively), 0- and 12-week HRLF (n = 35 and 23, respectively), 0- and 12-week LRHF (n = 21 and 22, respectively), 0- and 12-week HRHF (n = 45 and 28, respectively), and NC/naive rats (n = 30). The test was repeated five times, and the maximum grip strength (in grams) per trial for the reach limb is presented. Some rats used for behavioral data were not included in the biochemical or histological assays below, but were used for other experiments not included in this study. The person carrying out the testing was blinded to treatment. The naïve, 0- and 12-week LRHF and HRLF data, and portions of the 12-week HRHF grip strength data have been previously reported [55,58-60].

### Von Frey testing of forepaw mechanical sensation

Mechanical sensitivity was assayed as forepaw withdrawal behaviors to stimulation with von Frey filaments, as previously described [22] in the following rats: 0- and 12-week LRLF (n = 20 and 6, respectively), 0- and 12-week HRLF (n = 20 and 13, respectively), 0- and 12-week LRHF (n = 21 and 7, respectively), 0- and 12-week HRHF (n = 20 and 21, respectively), and NC/naive rats (n = 30). Some of these rats were used for other experiments not included in this study. The person carrying out the testing was blinded to treatment. Data from the preferred reach are presented. The 0- and 12-week LRHF data, and half of the HRHF forepaw mechanical sensation data, have been previously reported [22,55].

### Serum biochemical analyses

To study serum levels of inflammatory cytokines and biomarkers of bone turnover and cartilage degradation, animals were euthanized with a lethal overdose of sodium pentobarbital (i.p. injection, 120 mg/kg body weight) at 18 hours after completion of the final training or task session to avoid acute muscle activity induced changes in inflammatory cytokines. Blood was collected by cardiac puncture from: 0- and 12-week LRLF (n = 9 and 8, respectively), 0- and 12-week HRLF (n = 5 and 11, respectively), 0- and 12-week LRHF (n = 5 and 6, respectively), 0- and 12-week HRHF (n = 19 and 6, respectively), and NC rats (n = 13). Blood was prepared, serum collected and assayed using a customized multiplexed ELISA system (Aushon Searchlight Biosystem, Billerica, MA), as previously described [68] for three pro-inflammatory cytokines: tumor necrosis factor alpha (TNFalpha), interleukin 1-beta and interleukin 1-alpha (IL-1beta and IL-1alpha). Bone turnover markers were analyzed using commercially available ELISA kits: *a)* C1,2C (IBEX Technologies, Inc., Montreal, Quebec; measures types I and II collagen degradation fragments produced by collagenase cleavage); *b)* CTX1 (Immunodiagnostic systems, RatLaps; measures degradation fragments of c-terminal telopeptide of collagen type I released by osteoclast activity); and *c)* osteocalcin (Nordic Bioscience Diagnostics, Herlev, Denmark, Rat-MIDTM Osteocalcin; a protein produced by osteoblasts and a serum biomarker of bone formation). All serum samples were analyzed in duplicate in a blinded fashion, and data presented as ng or pg of analyte per ml of serum. Serum C1,2C and osteocalcin data has been previously reported for the 12-week HRHF rats only [62,69]. Serum inflammatory cytokine data have been previously reported for 12-week LRHF rats [55], 12-week HRHF rats [69], and 0-week and 12-week HRLF rats [59].

### Muscle, tendon and bone biochemical analyses

Forelimb flexor digitorum tendons and muscles were dissected off forelimb bones, and collected as flash frozen specimens, from subsets of the above rats: 0- and 12-week LRLF (n = 10 and 12, respectively), 0- and 12-week HRLF (n = 10 and 13, respectively), 0- and 12-week LRHF (n = 9 and 8, respectively), 0- and 12-week HRHF (n = 10 and 12, respectively), NC rats (n = 18) and FRC rats (n = 8). The radius and ulna, and the first row of carpal bones, were also collected together and flash frozen. Each tissue was homogenized separately per rat and assessed for TNFalpha, IL-1beta and IL-1alpha using commercially available ELISA kits (BioSourceTM, Invitrogen Life Sciences, CA) using previously described methods [68]. The inducible form of HSP70 (HSP72), a stress and repair protein, was analyzed using similar methods with a commercially available ELISA kit (Enzo Life Sciences, Farmingdale, NY) that has little cross-reactivity with other HSP70 family members, according to the manufacturers. Tendon levels of matrix metalloproteinase 2 (MMP2, a degradative enzyme), tissue transforming growth factor beta 1 (TGFB1, a repair and fibrogenic cytokine), and platelet derived growth factor ab and bb (PDGFab and PDGFab bb, repair proteins) were analyzed using a customized multiplexed ELISA system (Aushon Searchlight Biosystem, Billerica, MA). ELISA assay data (pg of cytokine protein and ng of HSP72) were normalized to μg total protein, determined using a bicinchoninic acid (BCA) protein assay kit. Inflammatory cytokine data for 12-week LRHF rats has been previously reported [55], as has 0- and 12-week HRHF data for muscle TNFalpha [69], and 12-week HRLF data for tendon TNFalpha and IL-1beta [61].

### Immunohistochemical and histomorphometric analyses of nerves, muscles and tendons

Following euthanasia by sodium pentobarbital (120 mg/kg body weight) and serum collection, subcohorts of animals were perfused transcardially with 4% paraformaldehyde in 0.1 M phosphate buffer (pH 7.4) at 18 hours after completion of the final training or task session. Tissues were immersion fixed for at least 24 hours. Then forearm musculotendinous tissues, with the median nerve intact, were dissected as a mass off forearm bones, and sectioned longitudinally as a soft tissue mass (*en bloc*), as described previously [20,58]. These *en bloc* tissue sections were stained using immunohistochemical methods for ED1, a marker of activated macrophages (MAB1435, Millipore, Billerica, MA), using previously described methods [70]. The median nerve was examined for the number of ED1+ macrophages per mm^2^, at the level of the wrist in preferred reach limbs in the following groups: 0- and 12-week LRLF (n = 4 and 6, respectively), 0- and 12-week HRLF (n = 6 and 9, respectively), 0- and 12-week LRHF (n = 6 each), 0- and 12-week HRHF (n = 6 each), and NC rats (n = 6), using previously described methods [70]. The person carrying out the computerized image analyses was blinded to treatment. The 0- and 12-week LRHF data and HRHF for ED1+ macrophages in the median nerve have been previously reported [22,55].

Flexor digitorum muscle and tendons were also collected from the above rats and examined for signs of histopathological changes in the *en bloc* soft tissue sections after staining with the ED1 antibody or hematoxylin and eosin (H&E). Muscles were defined as having microdamage by the presence of atrophied myofibers and the presence of ED1+ macrophages within myofibers (internal) in the same tissue section [27,43]. The number of myofibers with presence of ED1+ macrophages internal to the myofiber were counted in the mid-belly (widest) region of a cross-sectional slice of the flexor digitorum longus (region depicted in [58]), using a modification of previously described quantification methods and an image analysis system (Bioquant) connected to a Nikon E800, at three microscope field locations per rat [20]. No edema was observed in any muscle belly, so edema was not measureable. Tendons were scored at the level of the wrist using a modification of a semiquantitative scoring method (Bonar scale) for four factors: cell shape, collagen organization, cellularity, and amount of vascularization, in peritendon and endotendon, as described previously [58]. The person carrying out the computerized image analyses was blinded to treatment. Briefly, each factor was scored on a four point scale (0–3), with 0 being normal and 3 showing the most pathology; when summated, the total possible score would be 12. Each determination was made for each flexor digitorum tendon at the level of the wrist, at three microscope field locations per rat, in two to three separate sections per rat. Only the 12-week HRHF tendon data has been previously reported [58].

Spinal cords were also collected from the paraformaldehyde perfused animals. Lower cervical segments of the spinal cords were prepared, immunostained for substance P antibody (AB1566, Millipore), and quantified for percent area with Substance P immunostaining, as described previously [71], for: 0- and 12-week LRLF (n = 9 and 6, respectively), 0- and 12-week HRLF (n = 9 and 6, respectively), 0- and 12-week LRHF (n = 9 and 3, respectively), 0- and 12-week HRHF (n = 9 and 6, respectively), and NC rats (n = 9). The image analyses were carried out in a blinded fashion. The 12-week LRHF data has been previously reported [55].

### MicroCT analysis of distal radius

Radial bones collected from the above rats were used for micro-computerized tomography (microCT) analysis: 12-week LRLF (n = 6), 12-week HRLF (n = 6), 12-week LRHF (n = 6), 12-week HRHF (n = 6), with results compared to food restricted control (FRC) rats (n = 6), rather than NC rats, in order to control for possible bone loss due to the food restriction. Analysis of the distal radial trabecular bone was performed according to recent guidelines [72]. A Skyscan 1172, 12 MPixel model, high resolution cone-beam microCT scanner (Skyscan, Ltd, Antwerp, Belgium) was used to scan a 6 mm length of distal radius, using the following settings: x-ray source spot size of 300 nm, camera pixel size of 8.91 μm, Al 0.5 mm filter, voltage of 59 kV, current of 167 μA, rotation step of 0.40^o^, frame averaging of 4, a ring artifact correction of 10, and a beam hardening correction of 60%. The image slices were reconstructed using cone-beam reconstruction software (Skyscan NRecon) based on the Feldkamp algorithm, a process that yielded 8 μm thick sections in the axial plane, for each radius and ulna. Morphological traits were assessed starting 1 mm proximal to the growth plates, and then extending proximally from this position for 1 mm (112 slices). The volume of interest for trabecular microarchitectural variables was bounded to a few pixels within the endocortical margin. An upper threshold of 255 and a lower threshold of 75 were used to delineate each pixel as bone or non-bone; simple global thresholding methods were used. Trabecular morphometric traits were computed from binarized images using direct 3D techniques that do not rely on prior assumptions on underlying structures: trabecular bone volume per total volume (BV/TV), mean trabecular thickness (Tb.Th.), mean trabecular number (Tb.N.), and mean trabecular separation (Tb.Sp.).

### Histomorphometric analyses of cartilage and bones

The above bones scanned for microCT were then used for bone and cartilage histomorphometry, as were bones from trained only rats: 0- and 12-week LRLF (n = 4 and 6, respectively), 0- and 12-week HRLF (n = 6 and 9, respectively), 0- and 12-week LRHF (n = 6 each), 0- and 12-week HRHF (n = 6 each), and FRC rats (n = 6). Bones were processed and embedded in methyl methacrylate (MMA) or paraffin, sectioned into 3 to 5 μm thick longitudinal sections (3 μm for MMA; 5 μm for paraffin) and mounted onto slides, as described previously [62,73]. Slides were stained with Goldner’s Trichrome for counting osteoblasts, or immunohistochemically for TRACP (Cline Zy-9C5; Zymed Laboratories Inc., South Francisco, CA, USA) and ED1 (MAB1435, clone ED1, Millipore, Billerica, MA) for counting osteoclasts (ED1 is a marker of osteoclasts, macrophages and their progenitors; only multinucleated Trap5/ED1+ cells were counted in this study), as described previously [21,74]. Numbers of cells per bone surface (N.Ob/BS and N.Oc/BS) were counted in the same region as assayed using microCT using a Nikon E800 microscope interfaced with a Q-Imaging digital camera, and an image analysis system (Bioquant Osteo 2012, v12.1). The person carrying out the histomorphometry was blinded to treatment.

Morphological changes in the distal articular cartilage of the radius of paraffin embedded and sectioned bones were assessed after staining with Safranin orange (O) and fast green. A modified Mankin scoring system was used, which was derived from three subscores: *a)* structure, *b)* cellular abnormalities, and *c)* matrix staining, as described previously [62]. Within each subscore there was a range of possible values. *a)* The structure subscore had 7 possible scores, with 0 being normal and 7 showing the most disorganization (chaotic structure, clusters, osteoclast activity, presence of subchondral cysts). *b)* The cellular abnormalities subscore had 4 grades: 0 = normal, 1 = hypercellularity, 2 = abnormal clusters, and 3 = hypocellularity. *c)* The matrix staining subscore had 5 grades, with 0 being normal and 4 have an absence of staining. The average for each of the subscores was summated; the total possible score would be 16. The person carrying out the computerized image analyses was blinded to treatment. The 12-week HRHF data has been previously reported [62].

### Statistical analyses

Prism 4 Graph Pad software was used for the statistical analyses. Two-way ANOVAs were used to determine the differences between groups, with the factors repetition and force. The Bonferroni post-hoc method for multiple comparisons was used and adjusted p-values are reported. After the Bonferroni adjustment, a p-value of <0.05 was considered statistically different. All data are expressed as mean ± standard error (SEM). For succinctness, p values for the 2-way ANOVA and posthoc analyses are shown in the figures.

## Results

### Mean voluntary reach performance (reach rate and voluntary grasp force)

Because the inherent nature of our task is voluntary, the rats tended to reach and pull more frequently than their target rates, although over-reaching did not lead to increased successful reaches, as shown in Table 1. For example, in week 12 of task performance, the mean (± SEM) of all reaches per min (both successful and unsuccessful) was: LRLF (3.38 ± 0.31), HRLF (5.04 ± 0.45), LRHF (5.79 ± 0.97), and HRHF (9.27 ± 0.64). However, the mean number of successful reaches (± SEM) per group in week 12 was: LRLF (1.39 ± 0.06), HRLF (2.06 ± 0.39), LRHF (1.46 ± 0.25), and HRHF (2.89 ± 0.46), which was lower than the total number of reaches per minute. Similar results were observed at the end of week 1 (Table 1). This suggests that the rats did not rely completely on the auditory prompts, and did not effectively learn that a food reward could only be obtained 2 or 4 times per minute (for the low repetition or high repetition tasks, respectively), despite more frequent or partial pulls on the lever/handle, or, that they could not maintain the target force levels during task performance and were overcompensating their reach rate in order to garner a food reward. LRLF and HRLF rats were able to meet their requirement of 15% maximum pulling force (MPF) across the weeks of task performance. However, the mean pulling force of LRHF and HRHF rats was 46.22 ± 6.24 (mean ± SEM) and 48.64 ± 0.90 (Table 1), respectively, lower than the target of 53% (Table 1), indicating that these rats were consistently unable to meet the high force requirements.

### Muscle, tendon and bone inflammatory cytokine responses

**Figure 1:**
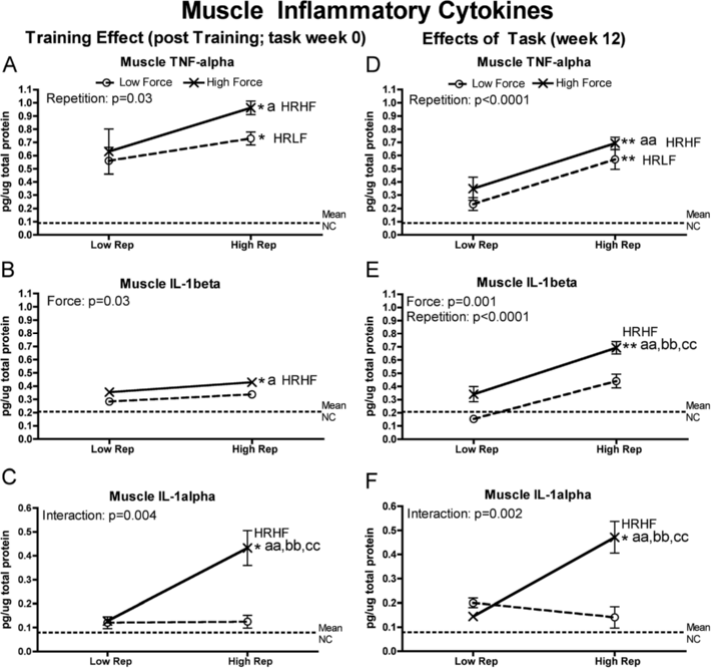
**Inflammatory cytokines levels in flexor digitorum muscles tested using ELISA in week 0 (immediately following the training period) or after performing either a LRLF, HRLF, LRHF and HRHF task for 12 weeks. (A & D)** Muscle TNFalpha in week 0 and 12. **(B & E)** Muscle IL-1beta in week 0 and 12. **(C & F)** Muscle IL-1alpha in week 0 and 12. Symbols: a and aa: p < 0.05 and p < 0.01, compared to LRLF rats; bb: p < 0.01, compared to HRLF rats; cc: p < 0.01, compared to LRHF rats; * and **: p < 0.05 and p < 0.01, compared to normal controls (NC) rats (indicated by dashed line). Mean and SEM are shown.

**Figure 2:**
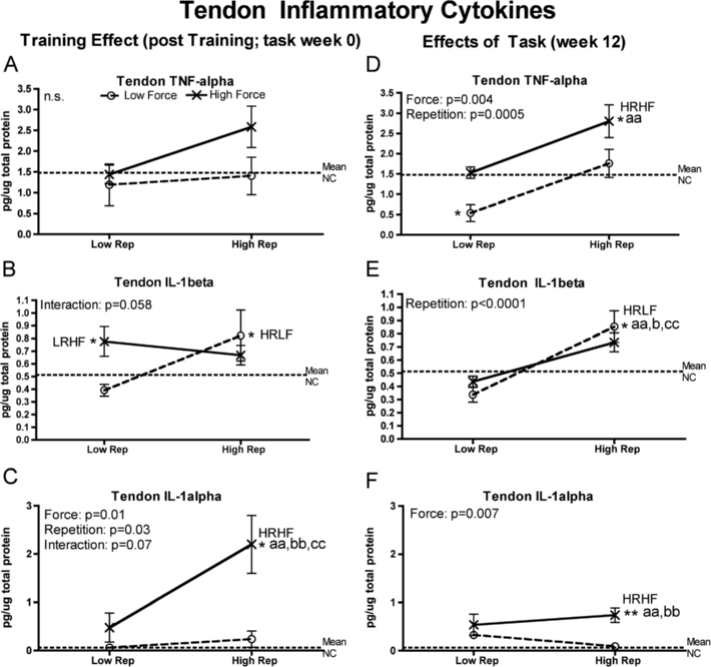
**Inflammatory cytokines levels in flexor digitorum tendons tested using ELISA in week 0 (immediately following the training period) or after performing either a low repetition LRLF, HRLF, LRHF and HRHF task for 12 weeks. (A & D)** Tendon TNFalpha in week 0 and 12. **(B & E)** Tendon IL-1beta in week 0 and 12. **(C & F)** Tendon IL-1alpha in week 0 and 12. Symbols: a and aa: p < 0.05 and p < 0.01, compared to LRLF rats; bb: p < 0.01, compared to HRLF rats; cc: p < 0.01, compared to LRHF rats; * and **: p < 0.05 and p < 0.01, compared to normal controls (NC) rats (indicated by dashed line). Mean and SEM are shown.

**Figure 3:**
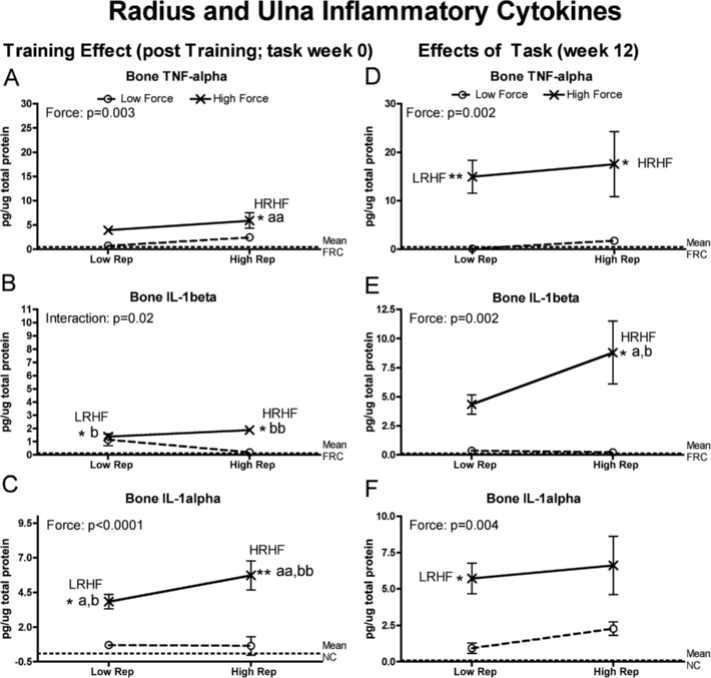
**Inflammatory cytokines levels in radial and ulnar bones, and first row of carpal bones, tested using ELISA in week 0 (immediately following the training period) or after performing either a LRLF, HRLF, LRHF and HRHF task for 12 weeks. (A & D)** Bone TNFalpha in week 0 and 12. **(B & E)** Bone IL-1beta in week 0 and 12. **(C & F)** Bone IL-1alpha in week 0 and 12. Symbols: a and aa: p < 0.05 and p < 0.01, compared to LRLF rats; b and bb: p < 0.05 and p < 0.01, compared to HRLF rats; * and **: p < 0.05 and p < 0.01, compared to food restricted controls (FRC) rats (indicated by dashed line). Mean and SEM are shown.

**Figure 4:**
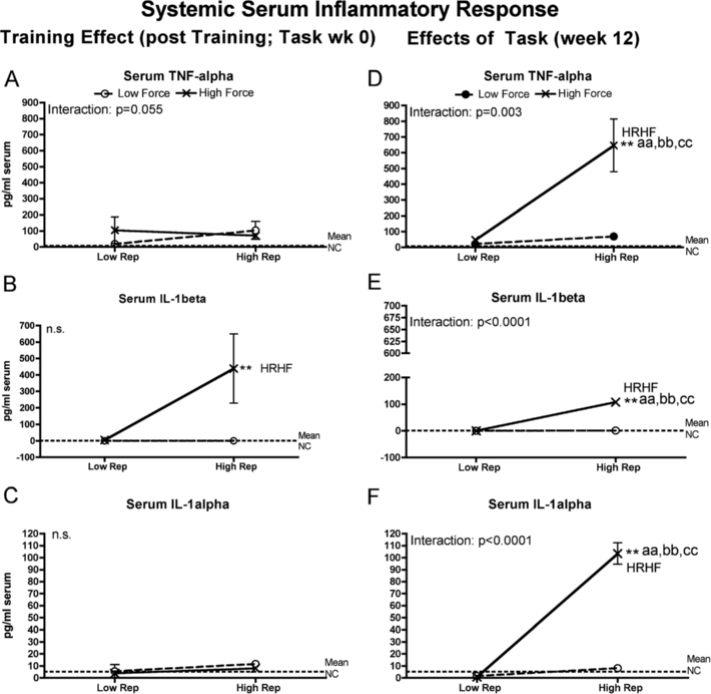
**Serum inflammatory cytokine levels tested using ELISA in week 0 (immediately following the training period) or after performing either a low repetition low force (LRLF), high repetition low force (HRLF), low repetition high force (LRHF) and high repetition high force (HRHF) task for 12 weeks. (A & E)** Tumor necrosis factor alpha (TNF-alpha) in weeks 0 and 12. **(B & F)** Interleukin 1-beta (IL-1beta) in weeks 0 and 12. **(C & D)** IL-1alpha in weeks 0 and 12. Symbols: aa: p < 0.01, compared to LRLF rats; bb: p < 0.01, compared to HRLF rats; cc: p < 0.01, compared to LRHF rats; **: p < 0.01, compared to normal controls (NC) rats (mean values of NC rats are indicated by dashed line). Mean and SEM are shown.

Since tissue injury increases production of inflammatory cytokines (as reviewed in [35,41,42]), we examined forelimb tissues for levels of TNF-alpha, IL-1alpha and IL-1beta. Flexor digitorum muscles and tendons, and forelimb bones (radius and ulna, and first row of carpal bones), had significantly increased inflammatory cytokines at the end of training (week 0) and at 12 weeks of task performance. They were particularly increased HRHF rat tissues, although HRLF rats had increased muscle TNF-alpha and tendon IL-1beta, and LRHF rats had increases in bones (Figures 1, 2 and 3). As described in detail below, force x repetition interaction effects were observed for 0- and 12-week muscles (IL-1alpha), 0-week tendons (trends only for IL-1beta and IL-1alpha), and 0-week bones (IL-1beta) (Figures 1C,F; 3B,C and 4B). Several individual effects from repetition and force were also observed, with the greatest increases in HRHF rat tissues.

In the muscles, a force x repetition interaction effect was observed for 0-and 12-week IL-1alpha (Figure 1C,F), individual effects from both repetition and force for 12-week IL-1beta (Figure 1E), and repetition or force effects for the remaining, with HRHF rat muscles having the highest levels than in the groups. Specifically, at the end of training (week 0), HRHF rat muscles had increased muscle TNF-alpha and IL-1beta, compared to NC and LRLF rats, and increased IL-1alpha, compared to the other groups (Figure 1A-C). HRLF rat muscles had increased TNF-alpha, compared to NC (Figure 1A). By week 12, HRHF rat muscles had increased TNF-alpha, compared to NC and LRLF rats, and increased IL-1alpha and beta, compared to the other groups (Figure 1D-F). 12-week HRLF rat muscles had increased TNF-alpha, compared to NC (Figure 1A).

In tendons, only trends for force x repetition interaction effects were observed in week 0 for IL-1beta and alpha (Figure 2B,C). However, individual effects from both repetition and force were observed for IL-1alpha in week 0, and TNF-alpha in week 12 (Figure 2C,D), repetition affected 12-week IL-1beta, and force affected 12-week IL-1alpha (Figure 2E,F). Specifically, after training (week 0), IL-1beta increased in LRHF and HRLF rat tendons, compared to NC (Figure 2B), and IL-1alpha increased in 0-week HRHF rat tendons, compared to the other groups (Figure 2C), By week 12, TNF-alpha increased in HRHF rat tendons, compared to NC and LRLF rats, but decreased in LRLF rat tendons, compared to NC (Figure 2D). The 12-week HRLF rat tendons had increased IL-1beta, compared to the other groups (Figure 2E), while 12-week HRHF tendons had increased IL-1alpha, compared to NC, LRLF and HRLF rats (Figure 2F).

In forelimb bones (radius and ulna bones, and first row of carpal bones), although one force x repetition interaction effect was observed, that of IL-1beta in week 0 (Figure 3B). Effects of force only was observed otherwise (Figure 3A,C-F). Specifically, after training (week 0), increases were seen for TNFalpha in HRHF bones, compared to NC and LRLF rats (Figure 3A); IL-1beta in LRHF and HRLF bones, compared to NC and LRLF rats (Figure 3B); and IL-1alpha in LRHF and HRHF bones, compared to NC, LRLF and HRLF rats (Figure 3C). By week 12, increases were seen for TNF-alpha in LRHF and HRHF bones, compared to NC rats (Figure 4D); IL-1beta in HRHF bones, compared to NC, LRLF and HRLF rats (Figure 3E); and IL-1alpha in LRHF bones, compared to NC rats (Figure 3F).

### Serum inflammatory cytokine responses

In order to determine if the increased tissue cytokines were detectable systemically, we next assayed serum for these same inflammatory cytokines. Levels of several inflammatory cytokines in serum showed force x repetition interactions with task performance, with HRHF rats having the highest levels (Figure 4). Specifically, analysis of serum after training (week 0) revealed force x repetition interactions for TNF-alpha that neared but did not significance (Figure 4A), and increased IL-1beta in HRHF rat serum, compared to NC rats (although no significant effect from force or repetition) (Figure 4B). By week 12, significant force x repetition interactions were observed for serum TNF-alpha, IL-1beta, and IL-1alpha, with high increases in HRHF rats, compared to the other groups (Figure 4D-F).

### Neural tissue responses (peripheral and central)

**Figure 5:**
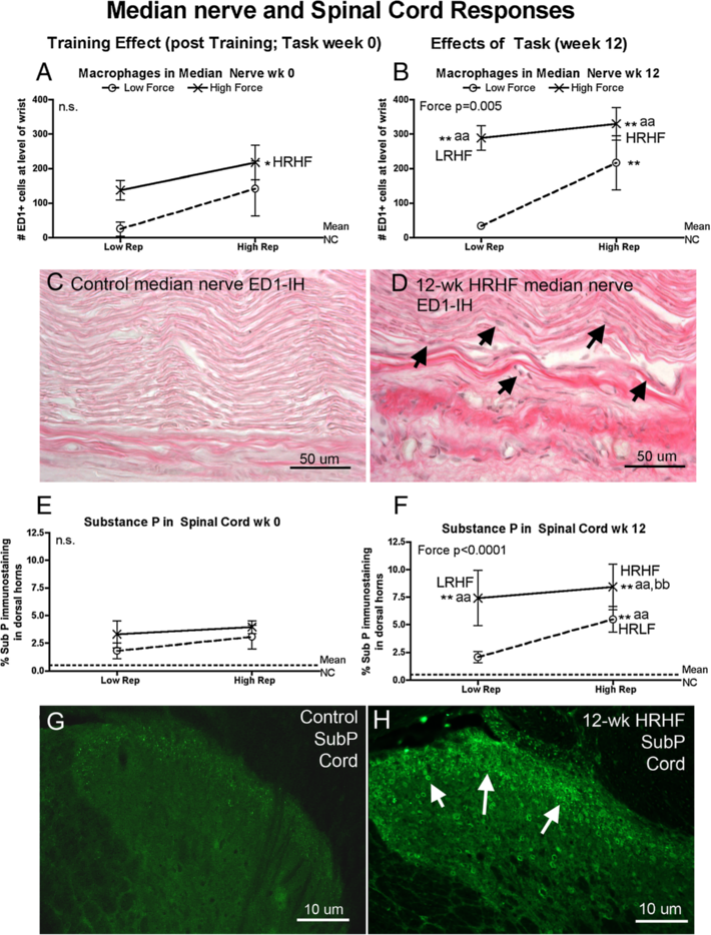
**Peripheral and central neural responses examined using quantitative immunohistochemical methods in week 0 (immediately following the training period) or after performing either a LRLF, HRLF, LRHF and HRHF task for 12 weeks. (A & B)** Mean number of ED1-immunoreactive (ED1-IH) macrophages in the median nerve at the level of the wrist, in week 0 and 12. **(C & D)** Representative photos of the median nerve at the level of the wrist, showing an absence of ED1-IH macrophages in a NC rat, but increased macrophages (stained black; arrows indicate a few) in a 12-wk HRHF rat. **(E & F)** Percent area with substance P immunoreactivity in the dorsal horns of lower cervical spinal cord segments, in week 0 and 12. Data for upper lamina (I and II) of the dorsal horns are presented. **(G & H)** Representative photos of the dorsal horn of lower cervical spinal cord segments, showing only low grade increases of substance P (SubP) immunoreactivity in a NC rat, but increased SubP in the upper lamina (arrows) in a 12-wk HRHF rat. Symbols: aa: p < 0.01, compared to LRLF rats; bb: p < 0.01, compared to HRLF rats; **: p < 0.01, compared to normal controls (NC) rats (indicated by dashed line). Mean and SEM are shown. Scale bars are as indicated.

Neuritis, in the form of increased activated macrophages in the median nerve at the wrist, was affected mainly by force at 12-weeks of task performance (Figure 5B). Specifically, increased macrophages were observed in 0-week HRHF, 12-week HRLF, 12-week LRHF rats, and 12-week HRHF, compared to NC rats (Figure 5A,B), and in 12-week LRHF and HRHF rats, compared to 12-week LRLF rats (Figure 5B). Figure 5D shows an increase of ED1-immunoreactive (ED1-IH; activated) macrophages in intraneural and extraneural regions of the median nerve at the level of the wrist in 12-week HRHF rats (arrows indicate representative macrophages), compared to an absence in NC rats (Figure 5C).

The main effect on substance P immunoreactivity (a nociceptor-related neurochemical) in the dorsal horns of cervical spinal cord segments was also force (Figure 5E-H). Specifically, no increase was observed in week 0 of any group. By week 12, increased substance P was observed in HRHF rats, compared to NC, LRLF and HRLF rats, and in 12-week HRLF and LRHF rats, compared to NC and LRLF rats (Figure 5D). Figure 5H shows increased substance P (SubP) in the dorsal horns of lower cervical spinal cord segments of 12-week HRHF rats, particularly in the upper lamina (arrows), compared to a NC rat (Figure 5G).

### Muscle subdegenerative, stress and repair responses

After training (0-week), flexor digitorum muscles showed no presence of macrophage infiltration into myofibers or edema that would be suggestive of myofiber degeneration [43]. No edema was observed in any muscle belly of 0-week rats.

**Figure 6:**
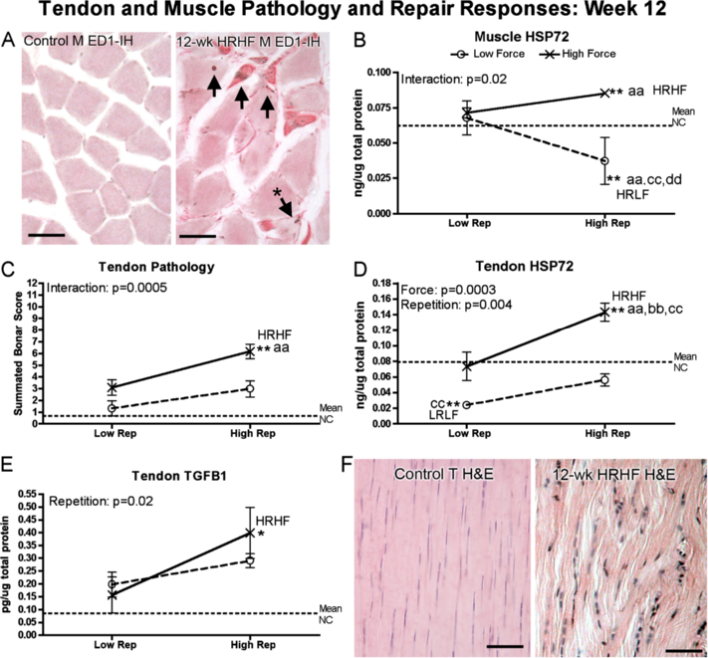
**Histopathology and production of repair proteins in flexor digitorum tendons and muscles after performance of either a LRLF, HRLF, LRHF and HRHF task for 12 weeks. (A)** Representative photos of ED1-immunoreactive (ED1-IH) macrophages in muscles (M) in a normal control rat and a 12-week HRHF rat. Arrows indicate ED1-IH macrophages; the arrow with the asterisk indicates a myofiber that is smaller than the others and that contains an ED1-IH cell within the myofiber, both indicative of a degenerating myofiber. Eosin counterstain (pink) shows increased collagen matrix between myofibers, indicative of fibrosis. **(B)** Inducible heat shock protein 72 (HSP72) in muscles. **(C)** Combined tendon pathology scores for cellularity, cell shape, and collagen organization (Bonar scoring system used in which 12 is the total score). **(D)** HSP72 in tendons. **(E)** Transforming growth factor beta 1 (TGFB1) in tendons. **(F)** Representative photos of H&E stained tendons showing elongated tenocytes and parallel collagen fibrils in a normal control endotendon, but rounded cells (tenocytes but also likely to include macrophages) and disrupted collagen fibrils in a 12-week HRHF endotendon. Symbols: aa: p < 0.01, compared to LRLF rats; bb: p < 0.01, compared to HRLF rats; **: p < 0.01, compared to normal controls (NC) rats (indicated by dashed line). Mean and SEM are shown. Scale bars = 50 micrometers.

By week 12 of task performance, flexor digitorum muscles showed significant force x repetition interactions in presence of macrophage infiltration into atrophied myofibers (photomicrograph shown in Figure 6A) and muscle levels of HSP72, a cell stress and repair related protein (Figure 6B). Specifically, a force x repetition interaction was observed for presence of ED1 cells within myofibers (p < 0.001). The 12-week HRHF flexor digitorum muscles had 17 ± 1.0 (mean ± SEM) myofibers with internal ED1-immunoreactive cells per mm^2^ at the level of the middle part of the flexor digitorum muscle belly, compared to 0.73 ± 0.02 in the LRHF group, and none within the HRLF or LRHF groups (graph not shown). Figure 6A right panel shows a representative photomicrograph from a 12-week HRHF rat muscle with subdegenerative type pathology in the form of a few myofibers with internal ED1-immunoreactive cells (arrows), an atrophied myofiber (asterisk points out a degenerating myofiber that is smaller and has a macrophage within the myofiber), and increased collagen deposition between myofibers (dark pink staining). No edema was observed in any muscle belly of 12-week rats. The 12-week HRHF rat muscles also had the highest levels of HSP72, while 12-week HRLF rat had the lowest, suggestive of reduced tissue stress in the 12-week HRLF muscles (Figure 6B).

### Tendons degenerative, stress and repair responses

After the training period (week 0 of task performance), no morphological signs of tendon pathology were observed in any 0-week rat groups (data not shown). However, flexor digitorum tendons showed a force x repetition interaction for HSP72 protein levels (p = 0.04). The 0-week HRHF flexor digitorum tendons had increased levels of HSP72 protein (ng/μg total protein) (0.11 ± 0.01 (mean ± SEM), p < 0.05 Bonferroni corrected post hoc p value), compared to 0.07 ± 0.009 in the LRHF tendons, and 0.07 ± 0.007 in the HRLF tendons, 0.07 ± 0.004 in the LRHF tendons, and 0.069 ± 0.003 in the NC rat tendons (graph not shown).

By week 12, flexor digitorum tendons showed a force x repetition interaction for tendon pathology (Figure 6C), individual effects from both force and repetition on HSP72 (Figure 6D), and effects of repetition on TGFB1 levels (a cytokine related to repair, although it may be fibrotic maladaptive repair [66,67]) (Figure 6D). Specifically, 12-week HRHF rat tendons showed increased pathology scores, compared to NC and LRLF rats (Figure 6C), the highest levels of HSP72 (Figure 6D), and increased TGFB1 (Figure 6E). HSP72 was decreased in 12-week LRLF tendons, compared to NC and LRHF rats (Figure 6D), suggestive of reduced tissue stress in these tendons despite continued performance of the task. Figure 6F right panel shows histological evidence of tendon pathology in 12-week HRHF rats as increased rounded cells (including tenocytes) in the endotendon, and increased disorganization and separation of tendon fibrils, compared to NC rats (Figure 6F left panel), which contained only slender/elongated tenocytes and closely packed parallel fibrils. Tendon levels of MMP2, PDGFab and PDGFbb were not above NC values in any group (data not shown).

### Net bone resorption versus formation

**Figure 7:**
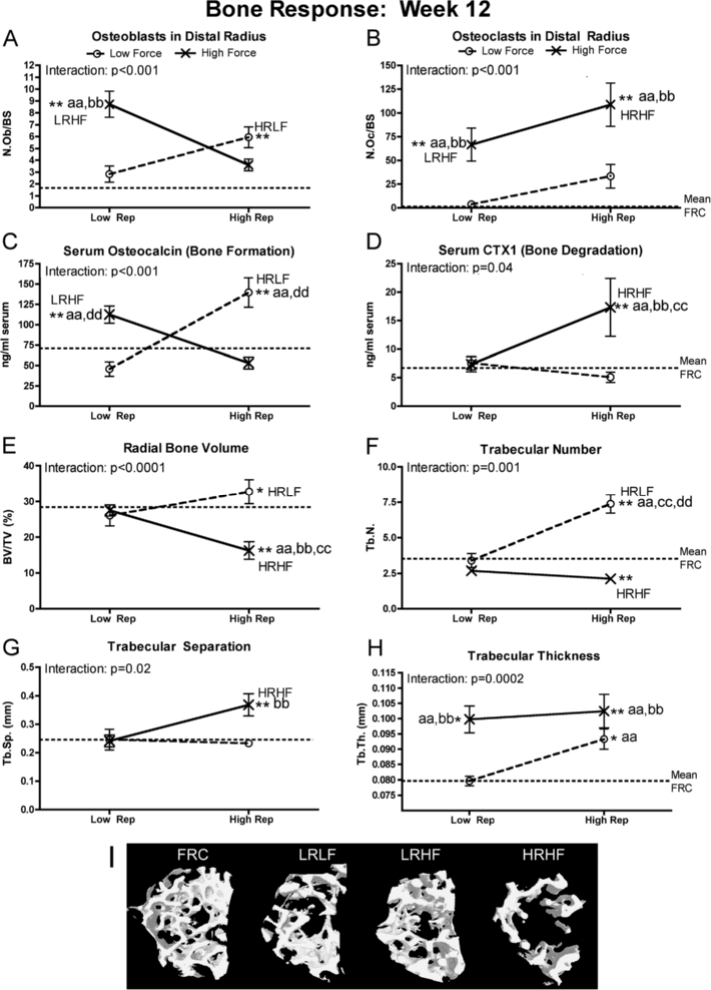
**Bone responses, assayed using histomorphometry, micro-computerized tomography and ELISA, after performance of either a LRLF, HRLF, LRHF and HRHF task for 12 weeks. (A-B)** Number of osteoblast and osteoclasts, normalized by bone surface (N.Ob./BS and N.Oc/BS), assayed using histomorphometry. **(C-D)** Serum levels of CTX1 and osteocalcin, serum biomarkers of bone formation and bone resorption, respectively, assayed using ELISA. **(D-H)** Micro-computerized tomography (MicroCT) results for trabecular bone volume (BV/TV) of trabecular bone located in the distal radial metaphysis, as well as trabecular number (Tb.N), trabecular separation (Tb.Sp.) and trabecular thickness (Tb.Th). **(I)** Representative transaxial views of the trabecular region of the distal radial metaphysis, captured using microCT scanning and reconstruction and then 3-D rendering, showing a significant loss of trabeculae in this region in 12-week HRHF rats, but not in FRC or 12-week LRHF rats. This region shown was used to generate the BV/TV data shown in panel G. Symbols: aa: p < 0.01, compared to LRLF rats; bb: p < 0.01, compared to HRLF rats; dd: p < 0.01, compared to HRHF rats; **: p < 0.01, compared to food restricted control (FRC) rats (mean FRC values are indicated by dashed line). Mean and SEM are shown.

Significant force x repetition interaction effects were observed for each bone morphological attribute and serum biomarker of bone turnover analyzed at 12 weeks of task performance (Figure 7). Several indicators of increased bone resorption were evident in 12-week HRHF rats (Figure 7B,D-G), while 12-week LRHF and HRLF rat had indicators of increased bone formation and adaptation (Figure 7A,C,E,F,H). Specifically, 12-week HRHF rats had several indices of net bone resorption in serum and in distal radial trabecular region, including increased osteoclast numbers, compared to NC, LRLF and HRLF rats (Figure 7B); increased serum CTX1 (a key serum biomarker of bone degradation), compared to the other groups (Figure 7D); decreased trabecular bone volume, compared to the other groups (Figure 7E); decreased trabecular number, compared to NC rats (Figure 7F), and increased trabecular separation, compared to NC and HRLF rats (Figure 7G). In contrast, indices of bone formation were visible in 12-week LRHF and HRLF rats had several indices of net bone resorption in serum and in distal radial trabecular region, including increased osteoblast numbers (Figure 7A); increased osteocalcin (a key serum biomarker of bone formation), compared to FRC, LRLF and HRHF rats (Figure 7C); and increased trabecular thickness, compared to NC, LRLF and HRLF rats (Figure 7H). The 12-week HRLF rats had increased trabecular bone volume, compared to NC (Figure 7E) and increased trabecular numbers, compared to the other groups (Figure 7F), changes indicative of bone adaptation to the task. Interestingly, 12-week HRHF rats also showed increased trabecular thickness, compared to NC, LRLF and HRLF rats (Figure 7H), indicative of some bone adaptation in this group. Figure 7I shows microCT 3D renderings of the distal radial trabeculae (with cortical bone segmented away from the trabeculae) demonstrating a clear reduction of trabecular bone in 12-week HRHF rats, compared to FRC, LRLF and LRHF rats.

### Cartilage pathology

**Figure 8:**
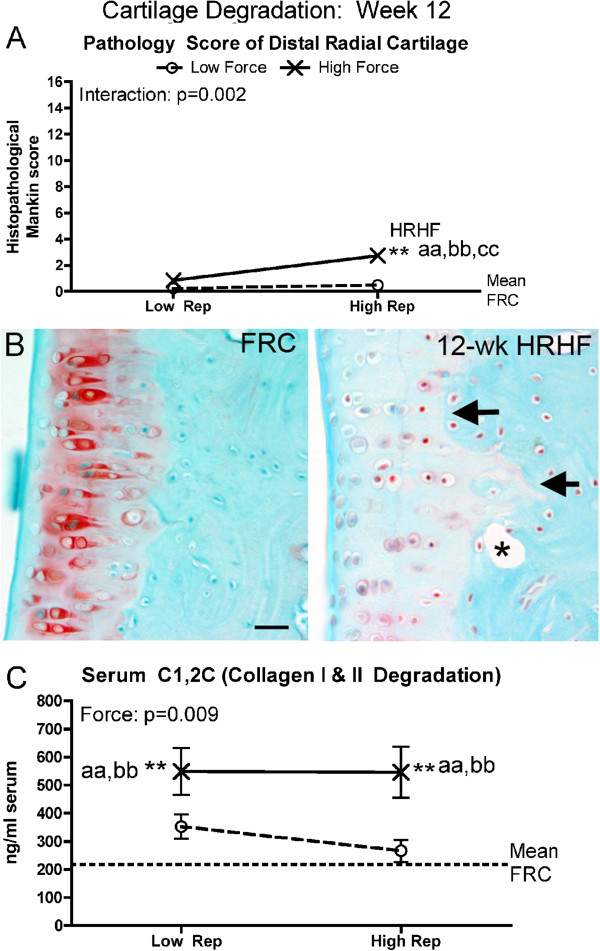
**Cartilage responses, assayed using histomorphometry and ELISA, after performance of either a LRLF, HRLF, LRHF and HRHF task for 12 weeks. (A)** Summated Mankin score results, a histopathological scoring system used to assay changes in the distal radial articular cartilage. **(B)** Representative photos of the distal radial articular cartilage showing a pathological loss of Safranin O (pink) staining, tidemark changes (arrows), and presence of a subchondral cyst (asterisk) in a 12-week HRHF rat; changes absent in a FRC rat (left part of panel). This region was used to generate the data shown in panel E. **(C)** Serum levels of C1,2C, assayed using ELISA. Symbols: aa: p < 0.01, compared to LRLF rats; bb: p < 0.01, compared to HRLF rats; dd: p < 0.01, compared to HRHF rats; **: p < 0.01, compared to food restricted control (FRC) rats (mean FRC values are indicated by dashed line). Mean and SEM are shown. Scale bar = 50 micrometer.

No evidence of cartilage pathology was observed in any of the 0-week trained only rat groups. By week 12, cartilage pathology was evident in HRHF rats (Figure 8). A significant interaction effect was observed for cartilage pathology (the Mankin score; Figure 8A), but a force effect only for serum C1,2C (a general marker of collagen type I and type II degradation) (Figure 8C). Specifically, in the distal radius articular cartilage, histological Mankin scoring showed increased pathology in 12-week HRHF rats (Figure 8A). Figure 8B right panel shows pathological changes in 12-week HRHF rats, including loss of proteoglycan content (reduced Safranin O, pink, staining), an altered tidemark region (arrows), and presence of subchondral cysts (asterisk). These attributes were not observed in FRC rat cartilage (Figure 8B left panel). Serum levels of C1,2C were increased in both 12-week LRHF and HRHF rats, compared to NC, LRLF and HRLF rats (Figure 8C).

### Sensorimotor functional responses

**Figure 9:**
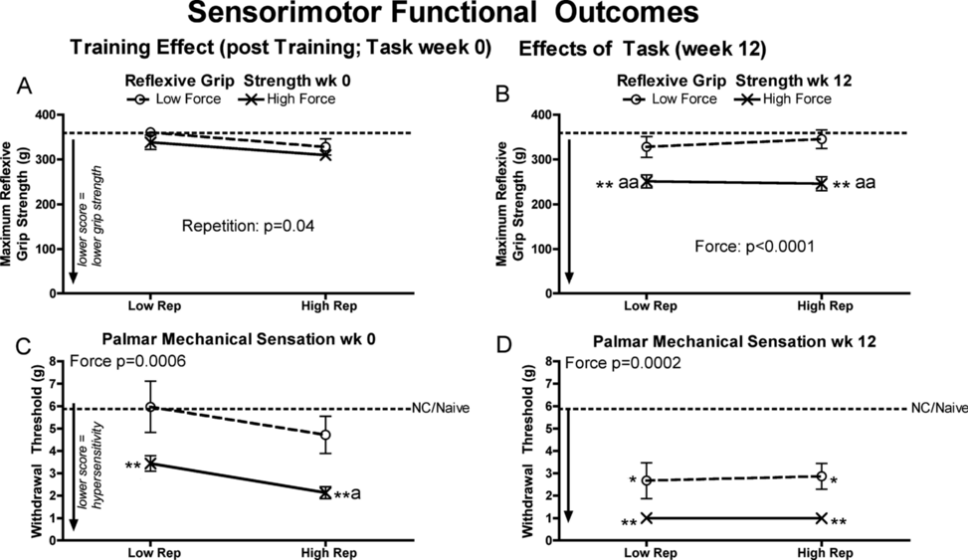
**Maximum reflexive grip strength and palmar mechanical sensation in the preferred reach limb, examined after performing either a LRLF, HRLF, LRHF and HRHF task for 12 weeks. (A & B)** Maximum reflexive grip strength in grams (g) in week 0 and week 12. **(C & D)** Palmar mechanical sensation, tested as withdrawal thresholds to mechanical stimulation with a series of von Frey hairs, in week 0 and week 12. Symbols: a and aa: p < 0.05 and p < 0.01, compared to LRLF rats; **: p < 0.01, compared to normal controls rat data combined with NC/naïve data (indicated by dashed line). Mean and SEM are shown.

Assessments of reflexive grip strength and withdrawal responses to mechanical stimulation of the glabrous forepaw showed these functions were affected by force or repetition, but not both (Figure 9). Specifically, reflexive grip strength was not reduced in 0-week rats, compared to NC/naïve levels (Figure 9A). By week 12, grip strength had reduced significantly in 12-week LRHF and HRHF rats, compared to NC/naïve levels and LRLF rats (Figure 9B). Forepaw withdrawal in response to mechanical stimulation altered so that smaller von Frey filaments elicited withdrawal responses (a sign of mechanical allodynia), than in NC rats or at the naïve testing point in 0-week LRHF rats, and in 0-week HRHF rats, compared to NC/naive and LRLF rats (Figure 9C). By week 12, LRHF and HRHF rats showed even greater mechanical allodynia, compared to NC/naïve levels (Figure 9D), although 12-week LRLF and HRLF rats also showed mechanical allodynia compared to NC/naïve levels.

## Discussion

Our aim was to investigate if serum and tissue inflammatory cytokines, degradation and injury markers, peripheral and central neural responses, and sensorimotor function exhibit force x repetition interaction responses in a rat model of work-related MSDs, similar to findings from epidemiological studies of humans with MSD risk [40]. We found that force and repetition had significant interactive effects on several serum inflammatory cytokines (TNF-alpha, IL-1beta and IL-1alpha), muscle IL-1alpha, bone IL-1beta, a muscle biomarker of stress (HSP72), presence of muscle, tendon and articular cartilage microdamage/pathology, bone volume density and morphometry in the distal radial trabeculae, and serum biomarkers of bone resorption and formation (CTXI and osteocalcin, respectively). On the other hand, repetition level influenced several inflammatory cytokines in muscles and tendons, and tendon stress and repair proteins (HSP72 and TGFB1). Force level was highly influential on bone inflammatory cytokine levels, macrophages in the median nerve, substance P in spinal cord dorsal horns, serum C1,2C (a biomarker of collagen type I and II degradation), grip strength and forepaw mechanical sensation. In most cases, performance of the moderate demand tasks (LRHF and HRLF) induced tissue changes indicative of reduced cellular stress and adaptation, while the HRHF task induced the greatest change from control levels and the most tissue degradation, as hypothesized by tissue tolerance [41], and tissue adaptation [46,52,53], and fatigue failure processes [28,31,40,47-51].

### The training effect

The data demonstrate significant effects of force by week 0 on forepaw mechanical sensation, on serum and tissue inflammatory processes (serum, muscle, tendon, bone and nerve), and HSP72 in tendons, but no signs of tissue microdamage or degeneration. Mechanical allodynia (defined as a threshold withdrawal response to decreased mechanical stimulation than normal control or naïve levels) was evident by the end of training in only the high force groups (week 0 LRHF and HRHF rats) (Figure 9C), matching past results from our lab showing mechanical allodynia in 0-week HRHF rats [69]. This is likely the result of neuritis, evidenced by increased ED1+ macrophages within and surrounding the median nerve in the high force groups, particularly in 0-week HRHF rats (Figure 6A). Activated macrophages secrete a myriad of chemicals, including inflammatory cytokines known to cause cytotoxic injury to axonal cell membranes and to increase pain symptoms [75,76]. TNF-alpha, which is released by injured cells and macrophages [41], induces ongoing activity in nociceptors when applied to intact nerves [77]. Prophylactic treatment of rats training to learn the HRHF task with an anti-rat TNFalpha drug blocked the development of forepaw mechanical allodynia [69], supporting an inflammatory signaling component for this pain behavior. Regarding the increased inflammatory cytokines in tissues after training, the greatest increases were observed in 0-week HRHF rats. We postulate that these increases indicate the onset of tissue microdamage. Intense and frequent training to overload levels is known to induce systemic inflammatory responses through the release of pro-inflammatory cytokines from injured tissues [45,46]. The increased inflammatory cytokines then stimulate tissue adaptation, repair, resorption or injury [78-81], based on future events and superimposed processes in these tissues [41,42,53,82-84]. The increased HSP72 in 0-week HRHF tendons is consistent with prior findings of an increase in this inducible repair protein after 4.5 weeks of high intensity training [81], an increase that may help drive an early beneficial inflammatory response and then regenerative tissue repair [85,86]. Thus, the 4–6 weeks of training to learn the tasks did not result in tissue microdamage or degeneration, but did elicit some inflammatory responses and repair responses (the increased HSP72 in tendons), and an inflammation-related behavior (forepaw mechanical allodynia).

### Muscle and tendon responses at 12 weeks of task performance

We observed that muscles and tendons showed a mixture of force x repetition interactions, individual effects from force and/or repetition. Adaptative type changes were observed in musculotendinous tissues of 12-week HRLF and LRLF rats, while increased inflammatory cytokines and indices of microdamage were the greatest in 12-week HRHF rat muscle and tendons. These findings match those from chronic stretch-shortening contractions studies, in which skeletal muscle adaptation can occur if the muscle is able to compensate to the increased demands of an activity, but maladaptive changes if the muscle is not able to meet these demands [25-27].

The forearm musculotendinous tissues appear to be accommodating to the lower and moderate demand tasks. The lack of inflammatory cytokine response in 12-week LRLF and 12-week LRHF rat muscles and tendons (Figures 1 and 2), suggest that injury mechanisms, and therefore inflammatory responses, are not present in these tissues at this time point. While the 12-week HRLF rats show increased muscle TNF-alpha (Figure 1D), HSP72 was decreased (Figure 6B), suggesting that HRLF muscles had benefited from the prolonged performance of this low force regimen, and had acclimated to the stress of the task more than the other groups. The decrease in tendon TNF-alpha in 12-week LRLF rats, and the significant decline in their tendon HSP72 levels, compared to NC rats, also suggests that LRLF tendons have adapted to the task, were no longer in stress, and may be better acclimated to metabolic stress than even NC tendons. HRLF rat tendons also showed lower HSP72 levels than NC rats, although not significantly lower after the Bonferroni adjustments (Figure 6D). These findings combined suggest that prolonged activity at low force parameters may have activated a variety metabolic changes that allow tissues to handle more efficiently the potentially damaging changes occurring with the tasks, as suggested recently [54]. Thus, the need for the inducible HSP72 was reduced in HRLF muscles and LRLF tendons by 12 weeks of task performance, even compared to NC rats. That said, functional gains were not observed in the 12-week HRLF and LRLF rats (Figure 9D), as we might expect based on tissue adaptation hypotheses [46,84,87], exercise training findings [54], or stretch-shortening contraction studies [25-27]. This may be because our model is not an exercise training study designed to result in strength gains, or due to the presence of pain, as shown by Andersen et al. [88,89].

In contrast, flexor digitorum muscles and tendons were affected negatively by continued performance of the HRHF task, and partially by the HRLF task (Figures 1, 2 and 6). The greatest inflammatory responses, highest muscle and tendon levels of a cell/tissue stress protein (HSP72), and evidence of muscle and tendon microdamage in 12-week HRHF rats, matching hypothesized outcomes for fatigue failure in which only the highest demand tasks or loads result in tissue pathology [29,30,40,90]. The increased inflammatory cytokines in muscles and tendons of 12-week HRHF rats indicates that these tissues were unable to accommodate to this task, as does the presence of macrophages within myofibers [24,43,91]. The number of macrophages within myofibers in this study was considerably lower than previously described after chronic stretch-shortening contractions, activity that produced considerably more injury and functional losses [24,43], indicating we have subdegenerative changes only. Isometric contractions are not known to produce injury in muscles [24,43], which suggests that the rats are not performing pure isometric grasping movements during this operant reaching and handle-pulling task, but are altering their strategy to achieve their food rewards.

The inducible form of HSP70 (HSP72), a repair protein and tissue stress marker that confers protection against ischemia and preserve cellular functions [92], was increased in only 12-week HRHF muscles and tendons (Figure 6B,D). This increased indicates that cell and tissue stress was sufficient in muscle and tendon of only HRHF rats to elicit this potentially mitigating response to tissue stress and injury [85,93]. The increased HSP72 may help drive tissue repair [85,86], as discussed further below. These results are consistent with recent findings by Sjogaard et al. showing that repetitive stressful work increased inducible HSP72 in muscles, while prolonged exercise training decreased its basal levels [54], showing a difference between injury responses produced by the two different types of activities.

TGFB1 (a cytokine implicated in inflammatory processes, wound healing, and fibrosis [94]) increased in tendons of HRHF and HRLF rats (Figure 6E), indicating that a repair process has been activated, although it may be a maladaptive type repair. TGFB1 increases in muscles under conditions of overload and injury, and has been linked to the pathogenesis of tissue fibrosis [87,94-96]. We have shown increased TGFB1, connective tissue growth factor (another fibrogenic factor), collagen type I and connective tissue deposition in muscles of 9-week HRHF rat [66] (but not in tissues from rats performing a lower demand high repetition negligible force task for 9 weeks, showing a exposure dependency for these increases) [66]. Similar increases in fibrogenic proteins and histopathological evidence of fibrosis and pathology are present in forearm muscles, tendons, and nerves of 12-week HRHF rats [22,56,58,66], compared to rats performing a low repetition negligible force task [58]. In contrast, HRLF rat tendons and muscles do not show fibrogenic changes until 18 weeks of task performance [97]. Unpublished studies from our lab show that inflammatory processes also resolve in HRHF muscles by 18 weeks, although maladaptive fibrotic processes persist. Rempel et al. have shown that repetition rate or number of loading cycles is associated with increased tendon microtears in a dose–response pattern, and that these early microstructural changes in repetitively loaded tendons may initiate degenerative processes that lead to fibrotic and disruptive tissue changes [98-102]. Fibrogenic changes can be prevented in our model if treated early in their development with anti-inflammatory drugs [66], showing that earlier inflammatory processes are contributing to later developing fibrotic responses. Thus, the simultaneous increase of repair proteins and inflammatory cytokines in 12-week HRHF tissues, concomitant with tissue pathology, suggests that tissue adaptation processes are not keeping pace with tissue injury. Furthermore, the fibrotic changes, evident as increased collagen matrix (i.e. fascia) within and surrounding muscles, tendons and nerves (see Figure 6A and references [22,55-58]) may distort dynamic biomechanical properties and increase tissue strain due to adherence to adjacent structures, as postulated by Driscoll and Blyum [103].

### Bone degradative versus adaptative responses

Although inflammatory cytokines in the forelimb bones (radius and ulna, and first row of carpal bones) were effected mainly by force levels, with increases of several cytokines observed in both LRHF and HRHF rats (Figure 3), it was clear from the morphological studies that bones responded catabolically to the 12 weeks of HRHF loading, and anabolically to the LRHF and HRLF loading (Figure 7). Bone responds to loading along a continuum ranging from anabolism to catabolism, depending on the magnitude, frequency and duration of loading [17,104-108]. Repetitive loading conditions, such as in studies of rats running on treadmills, performing repetitive jumping, and repetitive reaching at high force loads, show that increasing the intensity of weight-bearing or muscle loading exercise/activities may be associated with diminishing returns in bone morphology, such as declines in bone mass and quality [15-18]. Since IL-1alpha/beta and TNF-alpha promote bone resorption and inhibit bone formation [78,79], it is highly plausible that they are contributing to the observed bone resorption. The lack of inflammatory cytokine and morphological changes in 12-week LRLF rat bones (Figure 3 and 7) indicates that LRLF loading is not high enough to stimulate either bone resorption or formation, as shown in previous studies examining bone loading [52,109,110].

With regard to bone catabolism, we observed increased osteoclasts, increased serum CTX1 (a biomarker of bone resorption released by osteoclast activity), decreased radial bone volume and trabecular numbers, and increased trabecular bone separation in 12-week HRHF rats, each indicative of bone resorption and catabolism (Figure 7). The increased serum CTX1 in these rats could be due to the affects of HRHF loading on all forelimb bones involved in performing the task (radius, ulna, carpal bones, humerus and scapula). These results extend our prior reports of increased serum Trap5b (a serum biomarker indicative of osteoclast numbers), decreased epiphyseal plate height and cortical bone thinning in 12-week HRHF rats [18,62]. The increased resorptive changes in bone morphometry may also be a general catabolic effect from the increased circulating levels of inflammatory TNF-alpha and IL-1beta (Figure 4), cytokines known to produce bone catabolism if elevated systemically [111]. Other studies using involuntary cyclical loading animal models show that fatigue loading of bone leads to bone matrix or cell disruption [28,112], and increased bone resorption leading to a net bone loss and enhanced bone fragility [113,114]. We hypothesize that longer work periods will lead to more bone catabolism in the HRHF rats, consistent with the fatigue-loading theory, but are still investigating that hypothesis.

We were pleased to see signs of bone formation in the form of increased osteoblasts and serum osteocalcin in 12-week HRLF and LRHF rat, and a small increase in radial bone volume, as well as increased trabecular number and thickness in 12-week HRLF rats (Figure 7). The LRHF rats also showed increased trabecular thickness (Figure 7H). These findings suggest that radial bones are adapting positively to the prolonged loading at LRHF and HRLF levels. This is supported by prior findings showing qualitative signs of cortical bone adaptation in 12-week high repetition negligible force rats [21]. We have also shown increased serum osteocalcin in 6-week HRHF rats, although it declined with continued task performance to 12 weeks [18,62,69]. These results matches findings from other labs using involuntary loading animal models that show that bone loaded below the bone fatigue threshold undergoes bone formation [52,109,115], especially if animals are allowed a rest period between bouts of loading [116,117]. We hypothesize that longer work periods will lead to even greater gains of bone in HRLF rats, and perhaps in LRHF rats, findings that would be consistent with bone adaptation hypotheses [109,110,117-119].

### Cartilage degradative responses in HRHF rats

Histological evidence of articular cartilage degradation in the radial bone showed a force x repetition interaction that was present only in the 12-week HRHF rats (Figure 8A,B). Repeated high force loads are known to induce focal microtrauma in cartilage [120,121]. Therefore, our findings for cartilage support the fatigue failure process at a focal microtrauma level in articular cartilage with prolonged loading and high repetition high force load. In contrast, serum C1,2C (a serum by-product of collagen type I and II degradation; collagen type II is found only in cartilage) increased with both high force tasks. We have shown that catabolic changes in cartilage are linked to inflammation in our model, so that when inflammatory cytokines are attenuated with anti-inflammatory drugs, cartilage integrity is preserved in rats that continued to perform the HRHF task [62,69]. This is consistent with studies showing that after cartilage microtrauma, there is an increase in inflammatory mediators, including inflammatory cytokines, in synovial fluid, which stimulate catabolic enzymes that breakdown articular cartilage matrix (reviewed in [120]). The increased serum C1,2C in both 12-week LRHF and HRHF rats indicates high force induced cartilage degradation, although this degradation may not be confined to the radial carpal joint. We have observed cartilage degeneration in carpal bones [62], but have not examined cartilage changes in other forearm joints.

### Systemic responses at week 12

By week 12, levels of serum inflammatory cytokines assayed were affected by the interaction of force and repetition, so that the highest levels were induced by 12-weeks of HRHF task performance (Figure 1). Interactions were also observed for biomarkers of bone formation in LRHF and HRLF rats (osteocalcin, Figure 8C) and bone degradation in HRHF rats (CTX1, Figure 8D). These serum responses may provide the best gauge of overall tissue inflammatory, and bone formation versus degradation responses [41,44,82]. The interactions in inflammatory cytokine levels were less pronounced in tissues than in serum (although bone morphology and its serum biomarkers showed consistent force x repetition interactions). This is presumably because the serum response is reflective of tissue responses in all involved tissues of the upper extremity and body, not just the forearm tissues examined in this study. Inflammatory cytokines are also physiological mediators, not merely indicators, of inflammatory processes, cytotoxity, cell injury and osteoclast activity (and therefore mediators of bone resorption) [78-80].

It is noteworthy that the specific pattern of interactions in levels of serum inflammatory cytokines and biomarkers of bone turnover were as predicted by the fatigue failure theory, despite the relatively small group sample sizes, with, for example, the LRLF task showing the lowest increases in serum inflammatory cytokines, and the HRHF task showing the highest. This matches our prior exposure-dependent findings for serum inflammatory cytokines in rats performing a high repetition negligible force versus a low repetition negligible force task (a food retrieval task) for 8 weeks [68], and higher levels in 6-week versus 0-week HRHF rats [69]. Several clinical studies have reported increased serum inflammatory cytokines in patients with short-term upper extremity MSDs [122-124]. For example, sera from patients with upper extremity MSDs for 3 months had increased TNF-alpha and IL-1beta, compared to asymptomatic subjects [122]. Video terminal operators using the equipment more than 20 hr/wk have higher serum TNF-alpha, than controls that spent less than 2 hr/day using the equipment [124]. However, in our rat model the serum inflammatory cytokine response can resolve to baseline levels in HRLF and LRHF rats, despite continued task performance for as long as 24 weeks, presumably due to down regulation by anti-inflammatory cytokines or adaptation of tissues to task demands [55,59,67]. Studies examining sera and tissues from patients at the time of surgical intervention show no increase in serum inflammatory cytokines, but increased tissue TGFB1, a fibrotic repair cytokine, and fibrotic histopathology in tendons and connective tissues of the forearm [125,126]. We have recently shown that most inflammatory cytokines resolve towards baseline levels in serum, muscle and tendons of rats performing the HRLF task for 18 and 24 weeks. The musculotendinous tissues so not show restorative repair, but a moderate fibrotic repair instead [67]. This fibrotic response was detectable in serum as increased serum levels of TGFB1, connective tissue growth factor, matrix metalloproteinase 2 (a collagenolytic gelatinase) and hydroxyproline (a marker of collagen synthesis) [67]. These results combined indicate that the serum inflammatory cytokine response follows the fatigue-failure theory during acute phases of less than or equal to 3 months, but may not as inflammation resolves and restorative or fibrotic repair ensues. The extent to which the bone degrades or adapts to the tasks at time points longer than 3 months, and if the serum biomarkers of bone turnover are similarly altered, is still under investigation in our lab.

### Neural responses at week 12

The neural tissue analytes examined at week 12 show that force was the primary effector. The median nerve was affected by continued performance of the high repetition and high force tasks (Figure 5B). Only the LRLF task induced no increase in macrophages in or surrounding the median nerve (or substance P in the spinal cord dorsal horns). The presence of macrophages in perineurial zones surrounding the median nerve is indicative of an inflammatory process in the nerve, termed neuritis [77,127]. Perineurial and epineurial thickening from increased fibrogenic proteins, increased collagen deposition, and increased fibroblasts are also present in these nerve sheaths [22,55,70], changes indicative of maladaptive fibrosis in the connective tissue “container” surrounding nerves [127,128]. The presence of macrophages within the median nerve itself of HRHF rats (and a few in LRHF rats) indicate that axonal degeneration or myelin sheath damage has occurred, since the role of macrophages within nerves is to remove fragmented myelin sheaths [129,130]. This latter finding is indicative of fatigue failure at a microdamage level in the nerves with high force loading and partially with high repetition loading. This is further supported by past findings of reduced electrophysiological function in median nerves of LRHF and HRHF rats, evidenced by 15% to 16% declines in nerve conduction velocity [22,55]. These declines are comparable to the criteria for abnormal median nerve conduction (equivalent to 9% and 24% slowing of NCV) in human studies [131].

The increased substance P in dorsal horns of cervical spinal cord segments of 12-week HRLF, LRHF and HRHF rats (Figure 5E-F) was likely induced by peripheral inflammatory processes in the median nerve, but also in the musculotendinous tissues. We have observed HRHF-induced increases in substance P in peripheral tendons and connective tissues [58]. Evidence for the involvement of the spinal cord in the pathology associated with peripheral nerve compression injuries and pain has been demonstrated [132,133]. Substance P plays a central role in nociceptor signaling in the spinal cord and central sensitization associated with pain behaviors [132,133]. While the spinal cord is not undergoing direct task-induced injury in this model, these findings indicate that repetitive high force tasks induce central neural responses associated with pain behaviors.

### Sensorimotor declines in 12-week task rats

Reflexive grip strength, the peak force that a rodent can generate in forelimbs, was affected significantly by continued performance of both high force tasks (Figure 9B). We used a reflexive assay of grip strength, in that when pulled gently by the tail, the rodent grasps instinctively at a bar attached to a force transducer of a grip strength meter in order to stop this involuntary backward movement. When the pulling force overcomes their grip strength, the rodent loses its grip on the bar. The grip strength meter then records and displays the peak pull-force achieved by the limb. Treatment of HRHF rats with ibuprofen or an anti-rat TNFalpha drug partially attenuated these reflexive grip strength declines and reduced muscle inflammatory cytokine levels [60,66,69], indicating that the decreased grip strength in LRHF and HRHF rats is at least partially a consequence of increased musclotendinous inflammatory cytokines. Declines in forelimb grip strength occur after injection of TNF into forearm muscles [134], leading others to propose grip strength declines as a sign of movement-related or muscle hyperalgesia in flexor digitorum muscles [135]. Baker et al. [24] have reported declines in isometric force production after stretch lengthening contractions, with inflammation and damage to myofibrils postulated as injury mechanisms. Since anti-inflammatory drugs only partially attenuate declines in reflexive grip strength in our model [60,69], the myofibril microdamage may be contributing to the decline in grip strength, consistent with the fatigue failure hypothesis. However, the amount of myofiber damage observed here are considerably less than observed in the study by Baker et al. [24]. Chronic musculoskeletal pain also has a considerable negative effect on motor performance, impairing the ability to swiftly activate muscles for the production of rapid force contractions, impairing agonist–antagonist muscle activation patterns, and disturbing muscle force steadiness [88,89]. Thus, it is likely that muscle inflammation, myofiber microdamage, and pain are contributing to declines in grip strength in the LRHF and HRHF rats.

The LRHF and HRHF rats were consistently unable to meet the target high force grasp requirement of 53% of maximum voluntary pulling loads as early as week 1 (the end of the first week of performance of the tasks for 2 hrs/day, 3 days/week) and in week 12 (Table 1). The reaching and grasping task used requires both isometric activity of muscles of the distal forearm and forepaw flexors for the grasping part of the task (although like not pure, as discussed earlier), and concentric muscle activity in forearm extensors and proximal muscles of the shoulder and elbow for the reaching and finger extensions (See Additional file 2 and Additional file 1: Figure S1). As such, it requires not only normal physiological functioning within muscles [24,43], but also in the nerves, spinal cord, and sensorimotor cortices for full control [22,55,57,58,60,136]. This task also requires acquisition of a new motor skill, as evidenced by the pre-training time (taking 6 weeks to learn the LRHF and HRHF tasks), and by findings of improved percent success to 42% by week 9 [60]. Ibuprofen treatment further improved the percent of successful reaches in 9-week HRHF rats to 50% (compared to 42% in untreated rats), and improved grasping force to target levels, showing that tissue inflammation was preventing the rats from reaching the target grasp force [60]. However, by week 12, voluntary grasping forces in HRHF + Ibuprofen rats were lower (an 8% decline from 9-weeks), and the number of successful reaches had dropped precipitously from 50% to 11% [60], even though tissue inflammation was dampened by the ibuprofen treatment [62]. This suggests that motor control problems are affecting voluntary grasping, findings supported by studies examining the ability to perform grasping tasks after motor cortical lesions and after repetitive task induced remapping of the motor cortex [136-139]. Lastly, since this task is motivated by food reward, and reward delivery requires an operant action, it is likely that agonist muscles and alternate muscle strategies are being recruited to compensate for inflammation and damage in the HRHF tissues so that the rats can still achieve a food reward. The grasp pull force is generated primarily by the preferred reach limb, but as we and others have shown in reaching and grasping tasks, the animal grasps the handle with one paw, and braces and pushes off the inner cage wall with the other contralateral limb (See Additional file 1: Figure S1 and [58,136,140]. The pushing activity in the contralateral limb may be one compensatory means to maintain the operant grasp-pull forces.

Mechanical allodynia was also clearly affected by the high force tasks with continued task performance (Figure 9D). The mechanical allodynia is likely a result of the increased nerve macrophages seen in the LRHF and HRHF median nerves (Figure 5B,D), since increased nerve macrophages are indicative of inflammatory neuritis [127], and are temporally related to mechanical allodynia in our model [22,57,70]. We have previously reported that mechanical allodynia is blocked by systemic anti-rat TNFalpha treatment in 6-week HRHF rats [69], further linking this pain behavior with inflammatory cytokine levels. In patient studies, serum levels of inflammatory cytokines correlate with symptoms of pain and weakness, and are even predictive of the severity of patient symptoms [122-124]. These findings combined suggest that the observed nerve and systemic inflammatory responses contribute to pain behaviors.

### Fatigue failure theory

The fatigue failure theory suggests that when musculoskeletal tissues are loaded at low force levels, the deformations experienced by the tissues would be expected to be “elastic” in nature, in which loaded tissues return to their original shape in a linear “spring-like” fashion after the force causing the original deformation is removed. Thus, tissue damage would build up relatively slowly, if at all, as long as forces stay low. As forces and consequent stress on affected tissues increase, tissues may be deformed enough to reach their “elastic” limit, where the material may start to exhibit an inability to return to its original configuration. Such loading would, at some point, be likely to produce tendon inflammation, microtears or disruption [29,30,48], bone microscopic damage (e.g. increased resorption spaces and microcracks, which are small linear or elliptical cracks between osteons [23,28,112]); cartilage tidemark changes, microcracks and subchondral resorptive lesions [49,120,141]; or diffuse tissue damage when tissues are exposed to additional high force loading cycles. The theory of fatigue loading as a mechanism of tissue injury is supported in biomaterials testing of cadaveric materials, such as spine motion segments [142] and tendons [48,50,51,143]. In vivo studies show presence of kinked tendon fiber deformations in tendons subjected to low-level loading [20,29,30,121], versus increased matrix disorganization in tendons subjected to high-level fatigue-loaded [29,30]. There is a body of literature showing increased trabecular bone resorption after prolonged cyclical loading, and enlarged resorption spaces and microcracks in bones after fatigue or cyclical loading [23,28,112,113], changes implicated in skeletal fragility and stress fractures [112]. Cartilage also shows fatigue failure changes, including subchondral bone lesions in humans and racehorses after repetitive, high impact trauma [141], and articular cartilage thinning in forepaw digits of rabbits repeated flexion at 1 Hz with a mean peak digit load of 0.42 N for 2 h per day for 60 cumulative hours [49].

**Figure 10:**
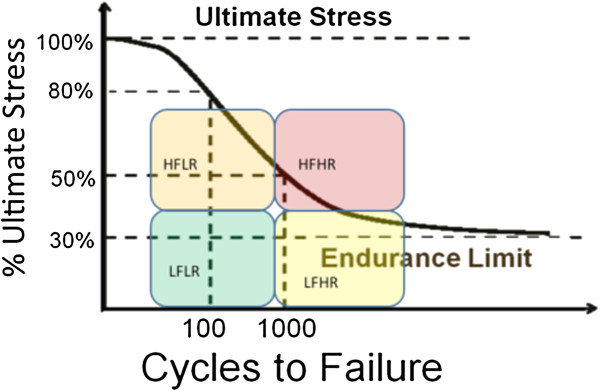
**Force x repetition quadrants superimposed on a fatigue failure curve.** Exposure of materials (tissues) to high force may result in failure within a fairly limited number of repetitions. As force exposure is decreased, tissues would be able to withstand many more repetitions before failure. This figure illustrates why a force-repetition interaction would be expected if tissues become damaged as the result of a fatigue failure process.

However, there is little indication in the literature that the implications of fatigue failure theory have been fully appreciated with respect to traditional occupational-related MSD risk factors [40]. The idea of fatigue failure predicts a specific pattern of interaction between MSD risk factors of force and repetition. Figure 10 illustrates the exponential fatigue failure (or S-N) curve overlaid with force-repetition quadrants, and indicates why a specific pattern of force x repetition interaction would be expected. As illustrated in this figure, high force tasks can be withstood for fewer cycles before failure, but as force is decreased many more cycles can be tolerated. In addition, there often exists an “endurance limit” below which a material can be repeatedly loaded without failing (or at least tolerate a very large number of repetitions before experiencing failure). For many materials, the endurance limit occurs at about 30% of the ultimate tensile strength of the material [144]. In vivo research in a sheep model suggests that in the most stressful locomotion conditions observed, the strain experienced by lateral digital extensor tendons peaks at approximately 25% of their predicted ultimate strength [144,145]. However, the demands of many occupational tasks have loads well above this level, leading to tissue damage. Of course, repair of biological tissues can be accomplished through the process of inflammation and remodeling, as the damage from repetitive loading does not exceed the ability of tissues to repair [41]. Unfortunately, the rate of biological repair can be rather modest; it is not unlikely that the deliberate pace of repair might be overwhelmed by continuing tissue damage from continued repetitive and forceful loading. This process may lead to a chronic inflammatory response and a cycle of re-injury, fibrosis, and tissue breakdown [41,82].

It might be noted that all measures of tissue damage (histopathology in muscles, tendons and cartilage, and morphometry in bone) demonstrated the expected force x repetition interaction predicted by fatigue failure theory. Serum inflammatory cytokine responses reflected this pattern as well, and likely represent a proportional inflammatory response based on the inflammation occurring in all involved body parts, not just in the forearm tissues examined. Not all of the tissue inflammatory cytokines showed the same pattern of response. One factor may be the inability of the rats to maintain the designated level of force production in the high force tasks throughout the study. This may have muted the responses of some measures to the point where an interaction was not apparent. In addition, some tissues or areas of tissues tested for certain markers may not have directly experienced injury, or the inflammatory peak may have already occurred prior (or subsequent) to tissue collection and cytokine measurement, leading to variable results for certain tissues and measures. There were several instances where an interactive tendency was observed for variables, but the interaction was not statistically significant. This may have been due to somewhat lesser statistical power associated with the “between subjects” design (necessary given the current research question) in combination with biological variability that may be substantial.

## Conclusions

A comprehensive examination of physiological, morphological and behavioral responses of exposure to varying levels of force and repetition in a unique rat model is described. The goal was to examine whether these physiological responses exhibit a force x repetition interaction indicative of a fatigue failure process in musculoskeletal tissues. Most of the systemic responses (serum inflammatory cytokines and serum bone degradation and formation markers) demonstrated such force x repetition interactions, as did presence of muscle, tendon and bone microdamage and pathology. These interactions were also observed by 12 weeks of task performance for muscle tissues for IL-1alpha and a stress marker (HSP72), but not for all cytokines in all tissues. Bone cytokine levels, for example, were affected by high force levels, as were functional measures and neural responses. It is clear from all of our findings combined that under certain conditions that beneficial adaptation can occur with prolonged performance of occupation-related tasks, with those conditions being: 1) a limited number of high force exertions and 2) sufficient time for the tissues to adaptively remodel. However, if the number of high force exertions are too many, or if sufficient rest between bouts of loading is not provided, then tissue inflammation and microdamage is the expected result. There may a tenuous balance between the two possible results. These findings support continued research on the fatigue failure hypothesis as a mechanism in the development of MSDs.

## Abbreviations

BV/TV: Bone volume density; bone volume per total volume; C1,2C: Serum biomarker of cartilage and collagen degradation; indicative of serum levels of types I and II collagen degradation fragments produced by collagenase cleavage; CTX-1: Serum biomarker of bone degradation; indicative of serum levels of degradation fragments of c-terminal telopeptide of collagen type I released by osteoclast activity; ED1: A marker of activated macrophages; ELISA: Enzyme linked immunosorbent assay; FRC: Food restricted control; LRLF: Low repetition low force; LRHF: Low repetition high force; HRLF: High force low repetition; HRHF: High repetition high force; HSP72: Heat shock protein 72, the inducible form of HSP70; IL-1: Interleukin 1; MicroCT: Micro computerized tomography; MSDs: Musculoskeletal disorder; MMP: Matrix metalloproteinase 2; NC: Normal control; Pax7: Paired box homeotic gene 7; PDGF: Platelet derived growth factor; SubP: Substance P; TGFB1: Transforming growth factor beta 1; TNFalpha: Tumor necrosis factor alpha.

## Competing interests

The authors declare that they have no competing interests.

## Authors’ contributions

MFB conceived of the study, directed its design and coordination, performed the statistical analysis, and drafted the manuscript. SG also co-conceived of the study, aided in its design and coordination, and co-drafted the manuscript. VSM performed part of the serum bone ELISA and microCT, and contributed to drafting the manuscript. MT conceived of the HSP72 part of the study, and helped to draft the manuscript. AEB-G helped to conceive of the study and its design and coordination, helped to draft the manuscript. All authors read and approved the final manuscript.

## Pre-publication history

The pre-publication history for this paper can be accessed here:

http://www.biomedcentral.com/1471-2474/14/303/prepub

## Supplementary Material

Additional file 1: Figure S1 Rat performing HRHF repetitive reaching task. (A) Rat awaits auditory stimulus with snout in portal. (B) Rat reaches for force handle with left forepaw; right forepaw used for postural support. (C) Viewed from top, rat grasps and isometrically pulls force handle attached to force transducer, until predetermined force threshold is reached and held for at least 50 ms. (D) Rat retrieves foot pellet reward by mouth from food trough. (E). Photo showing position of portal and light used for cueing. (F). Photo showing auditory clicker, position of handle external to portal and its attachment to a stationary force transducer, and mixture of grain based and banana flavored food pellets. Click here for file

Additional file 2 **Video file showing a rat performing HRHF repetitive reaching task, as described in Additional file **1**: Figure S1.** Click here for file
