# Supplementary figures and images for: The interaction of force and repetition on musculoskeletal and neural tissue responses and sensorimotor behavior in a rat model of work-related musculoskeletal disorders
Source: BMC Musculoskelet Disord. 2013 Oct 25;14:303. doi: 10.1186/1471-2474-14-303 (PMC3924406; doi:10.1186/1471-2474-14-303)

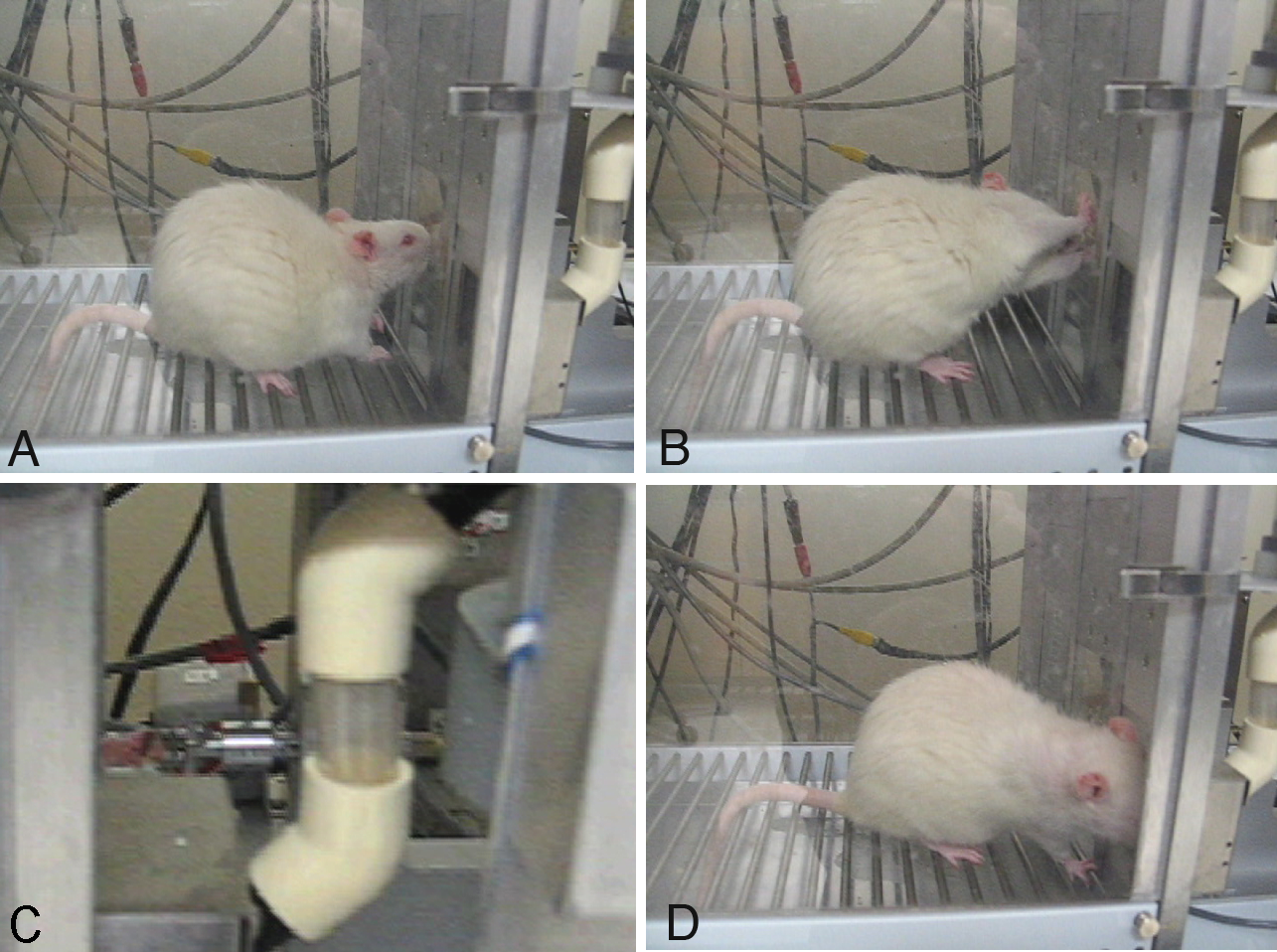

Supplement: Additional file 1: Figure S1 — Rat performing HRHF repetitive reaching task. (A) Rat awaits auditory stimulus with snout in portal. (B) Rat reaches for force handle with left forepaw; right forepaw used for postural support. (C) Viewed from top, rat grasps and isometrically pulls force handle attached to force transducer, until predetermined force threshold is reached and held for at least 50 ms. (D) Rat retrieves foot pellet reward by mouth from food trough. (E). Photo showing position of portal and light used for cueing. (F). Photo showing auditory clicker, position of handle external to portal and its attachment to a stationary force transducer, and mixture of grain based and banana flavored food pellets. [file 1471-2474-14-303-S1.tiff]
